# Structural Principles of Covalent Flavin Modification in Oxidoreductases

**DOI:** 10.1021/acs.biochem.6c00163

**Published:** 2026-04-27

**Authors:** John J. Tanner

**Affiliations:** Departments of Biochemistry and Chemistry, University of Missouri, Columbia, Missouri 65211, United States

**Keywords:** flavoenzymes, covalent inhibition, covalent modification, X-ray crystallography

## Abstract

Flavin-dependent oxidoreductases participate in a remarkably diverse array of biochemical transformations, enabled by the redox and covalent versatility of the isoalloxazine cofactor. The N5 and C4a atoms of the flavin are central to catalysis and, in selected cases, serve as loci of irreversible covalent modification. This review focuses on structurally validated examples of covalent inactivation of flavoenzymes in which the chemical nature of the flavin adduct has been established by X-ray crystallography, beginning with the historically important reversible N5 sulfite adduct and extending to mechanism-based irreversible inactivation. Mining of the Protein Data Bank reveals that covalent flavin modification occurs across multiple enzyme families, including monoamine oxidases, lysine-specific demethylase 1, proline dehydrogenase, spermine/polyamine oxidases, and cytokinin oxidase/dehydrogenase. These systems illustrate a limited but recurring set of structural outcomes, most commonly N5 alkylation or C4a alkylation, frequently accompanied by flavin reduction and characteristic butterfly bending of the isoalloxazine ring. A theme emerging from the structural record is the susceptibility of amine oxidases and dehydrogenases among covalently inactivated flavoenzymes. This prevalence reflects intrinsic mechanistic features of C–N bond oxidation—particularly iminium formation proximal to reduced flavin—that predispose these enzymes to irreversible flavin modification. In contrast, other flavoenzyme classes rarely generate electrophiles positioned for flavin attack, rendering stable covalent modification less common. By integrating structural, mechanistic, and inhibitor-design perspectives, this review highlights both the chemical disposition underlying covalent flavin inactivation and the constraints that shape its distribution across flavoprotein biochemistry.

## Graphical abstract

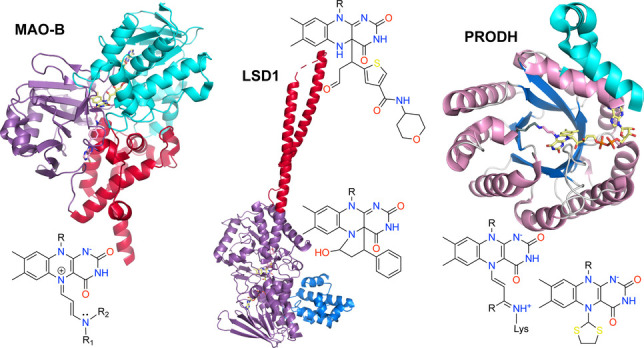

## Introduction

Flavoenzymes are distinguished by the presence of the eponymous cofactor in the active site. The isoalloxazine moiety of flavins is a reactive center that enables chemistry beyond the scope of the 20 amino acids. As many have written, the isoalloxazine is perhaps the most versatile of the redox-active cofactors in the cell.^1–4^ One aspect of this versatility is that the isoalloxazine can accept or donate either one electron at a time or a pair of electrons (as a hydride ion). This enables flavoproteins to serve as electron bridges between 1-electron and 2-electron biochemical processes. Another aspect is the large number and diversity of chemical reactions in which flavoenzymes participate, including both redox and nonredox reactions.^4–7^

Canonically, flavoenzymes catalyze oxidation–reduction reactions. In these enzymes, the isoalloxazine may adopt oxidized, one-electron-reduced (semiquinone), and two-electron-reduced states during the catalytic cycle (Scheme 1). These flavin redox states have distinctive spectroscopic signatures (Figure 1).^8^ Oxidized flavin has a yellow color, with strong absorbance bands near 360–380 and 440–460 nm. The one-electron reduced semiquinones are diagnostically different: the neutral, or “blue,” semiquinone (FADH·) shows a broad long-wavelength band in the ∼550–650 nm region, whereas the anionic, or “red,” semiquinone (FAD·^–^) is shifted to shorter wavelengths and typically absorbs more strongly in the ∼360–500 nm range. The two-electron reduced anion, FADH^–^, loses the intense band of the oxidized flavin at ∼450 nm and is dominated by near-UV absorption, with only weak visible absorbance that decays toward the baseline above ∼500 nm. In proteins, the exact maxima and intensities can shift, but these overall patterns are the key spectral signatures used to distinguish flavin redox states.

**Scheme 1:**
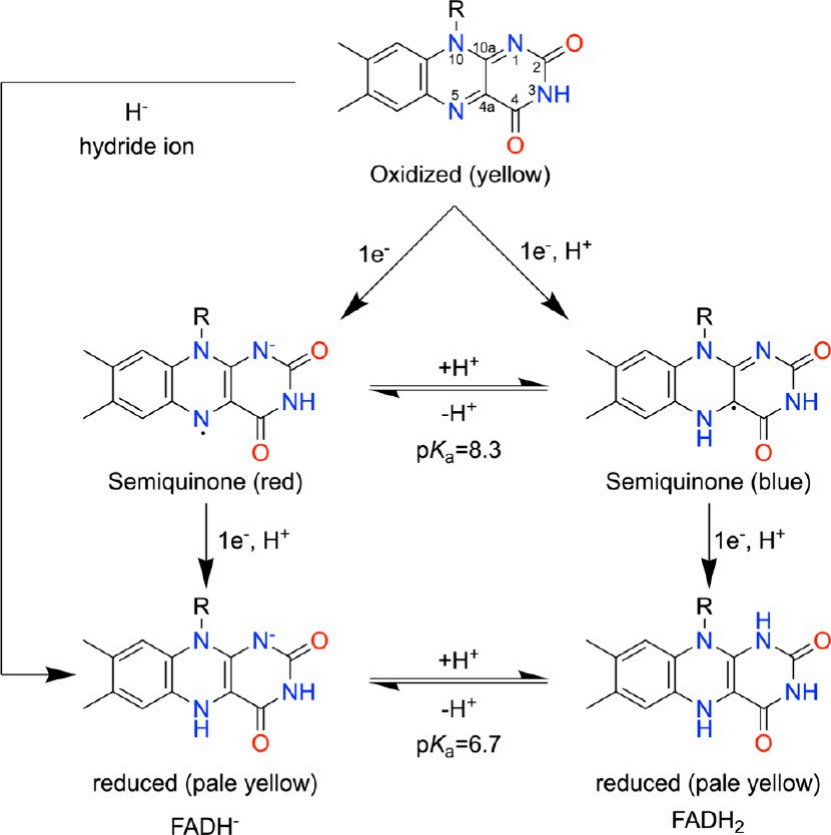
Flavin Redox States (Adapted From Crozier-Reabe and Moran^13^)­^a^

a The *si* Face of the Isoalloxazine Is Toward the Viewer.

**Figure 1:**
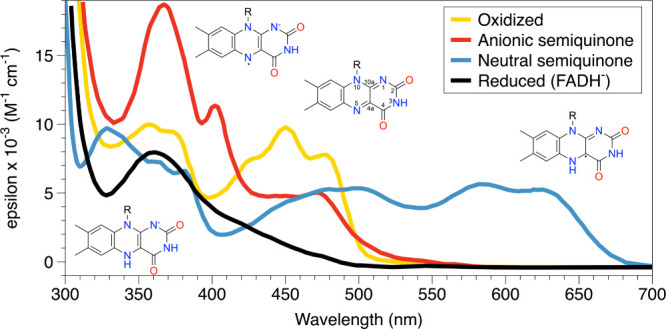
Spectra of the flavoprotein redox states. The data points used to make these curves were extracted from Figure 1 of Liu et al.^14^ using graphreader.com.

The N5 and C4a atoms of the isoalloxazine play crucial roles in redox biochemistry, with N5 being the primary hydride donor/acceptor. Also, in some flavoenzymes, these atoms are the loci for the formation of reactive intermediates. For example, the classical flavin-C4a-(hydro)­peroxide and the more recently discovered flavin-N5-(per)­oxide intermediates are the oxygenating reagents in flavin-dependent monooxygenases,^4,9–11^ and an N5 imine species appears in the mechanism of nitroalkane oxidase.^12^ The reactivity of the N5 and C4a atoms makes them attractive targets for covalent inactivators.

Not all flavoenzymes are oxidoreductases. Noncanonical flavoenzymes catalyze reactions with no net redox change. The protein environment in noncanonical flavoenzymes modulates the reactivity of the flavin to perform novel chemistry, usually featuring covalently modified flavin species as intermediates.^7,15^ For example, the first step in the mechanisms of UDP galactopyranose mutase^16^ and alkyl-dihydroxyacetone phosphate synthase^17^ is the reaction of the reduced flavin with the substrate to generate a transient imine-N5 adduct. Covalent inactivation of noncanonical flavoenzymes has been studied; one example is the covalent inactivation of isopentenyl diphosphate isomerase by 3-methylene-4-penten-1-yl diphosphate and 3-oxiranyl-3-buten-1-yl diphosphate, which results in modification of the C4a atom (pdb_00003b03, pdb_00003b04).^18^

This review focuses on structurally validated examples of covalent inactivation of flavin-dependent oxidoreductases, in which the chemical identity of the modified flavin has been established by X-ray crystallography. The surveyed research is mainly from the inhibitor discovery area and involves mostly intentional, but sometimes serendipitous, covalent inactivation of flavin-dependent oxidoreductases by synthetic molecules. By restricting the scope to crystallographically characterized adducts, we aim to distill general structural principles governing flavin reactivity rather than catalog spectroscopic or kinetic observations alone. Mining the Protein Data Bank (PDB) reveals that covalent flavin modification is both mechanistically constrained and unevenly distributed across flavoenzyme families, with amine oxidases representing a disproportionate fraction of known examples. Understanding why this distribution arises provides insight into not only inhibitor design but also the intrinsic chemical logic of flavin-dependent catalysis.

This review neglects certain aspects of covalently modified flavins. We do not cover the covalent attachment of flavins to amino acids that occur naturally in some enzymes (flavinylation),^19^ or the prenylated flavin in the Ubix/Ubid system.^2,20^ Nor do we consider the modified flavins that appear as reaction intermediates, such as C4a hydroxy- and peroxyflavin, N5-oxide, N5-peroxo, and N5-iminium, which have been expertly reviewed previously.^2,3^

## Mining the PDB for Covalently Inactivated Flavoenzymes

Because this review focuses on structural aspects of covalent inactivation of flavoenzymes, the PDB was searched for covalently modified flavins. Currently, the cheminformatic descriptions of covalently modified flavins in the PDB are inconsistent, which makes comprehensive cataloging somewhat challenging. In some structures, the modified flavin has the traditional residue name of FAD, FMN, FDA (reduced FAD), or FNR (reduced FMN), and the covalent modification has a different residue name. An example is the structure of MAO-B inactivated by pargyline (pdb_00001gos), where the modified flavin consists of an FAD residue bonded to residue NYP. In other structures, the PDB has assigned a unique residue name to the modified flavin. For example, the FAD covalently modified by 1,3-dithiolane in pdb_00007mya has the residue name UJJ. Thus, two different strategies were used to find covalently modified flavins in the PDB.

The first method was designed to find covalently modified flavins that retain the traditional PDB flavin residue names (FAD, FDA, FMN, and FNR). The database of structures was assembled using a Python script generated by ChatGPT 5 that downloaded PDB entries containing FAD, FDA, FMN, or FNR. The database contains 2916 FAD-containing structures, 145 with FDA, 1627 with FMN, and 55 with FNR. Covalently modified flavins were identified based on interatomic distance calculations using a script generated with the help of ChatGPT 5. For each PDB entry, the script calculated all interatomic distances between atoms in different residues within a given cutoff distance. A cutoff of 2.1 Å was used to capture likely covalent bonds. The list of distances was curated to remove peptide bonds and bonds to amino acids, which occur from flavinylation. The remaining hits were curated manually to identify covalently modified flavins relevant to enzyme inactivation.

A different approach was used to find covalently modified flavins that have unique residue names. These entries were found using the “Similar Ligands (Quick Screen)” searching tool of the PDB. Several flavin-containing ligands were used as queries, including FAD, FDA, FNR, FMN, 12F, 2PF, 6YU, A1BC6, A1BI4, A1H8N, A1IG2, D3U, D51, D52, D69, D70, D73, F2N, FA8, FA9, FAA, FAB, FAJ, FCG, FNK, HUF, KWM, KXM, P5F, SFD, SV9, UJG, UJJ, XB3, XB6, XF6, XHT, XRK, XZQ, Y0Z, Y66, Y9K, YAF, YAO, and ZSI. From these searches, a nonredundant list of residue names was compiled and then used to find PDB entries containing those residues. The resulting list of PDB entries was curated manually to identify those relevant to enzyme inactivation. The curated list is provided as a spreadsheet in the Supporting Materials.

## Sulfite: A Nonspecific Inactivator of Flavoenzymes

The sulfite ion (SO_3_^2–^) is a nonspecific reversible covalent inhibitor of some flavoenzymes. Early studies of Muller and Massey showed that sulfite forms a reversible covalent adduct with the N5 atom of enzyme-free model flavins.^21^ The adduct formation reaction is reversible in the pH range >2. Absorbance spectroscopy of the sulfite adduct showed that the flavin is fully reduced. The mechanism of the reaction is straightforward. As Muller and Massey wrote, oxidized flavin is electron-deficient, while sulfite is a potent nucleophile. Thus, the reaction occurs by nucleophilic attack of the S atom of sulfite on N5 of the flavin, resulting in a reversible covalent bond (Figure 2A). Sulfite differs fundamentally from the other inhibitors described here in that it does not require activation by the flavin. Nevertheless, these early studies clearly established that the isoalloxazine is not just a redox center, but a chemically reactive heterocycle. This reactivity would be exploited in the design of mechanism-based covalent inactivators, as described in subsequent sections.

**Figure 2:**
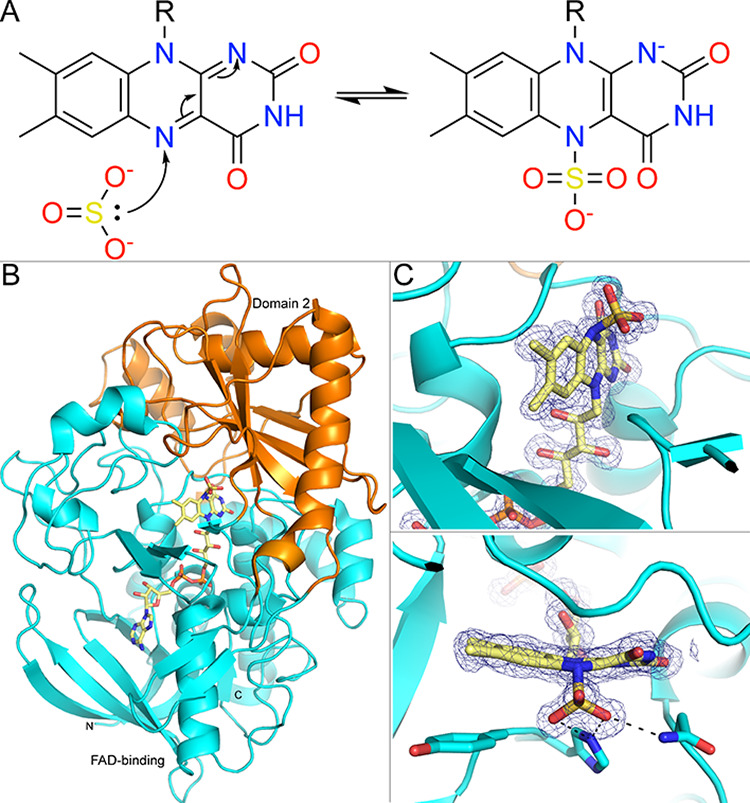
Covalent modification of flavoenzymes by sulfite. (A) Mechanism of reversible covalent modification. (B) Structure of the covalent complex of cholesterol oxidase with sulfite (pdb_00004u2l). The protein is colored by domains. (C) Electron density for the FAD-sulfite adduct (2*F*_0_-*F*_c_, 1σ). Side chains that interact with the SO_3_^–^ group are shown.

The regiochemistry of the sulfite reaction was validated by X-ray crystallography of flavoenzymes. The structures of the Sulfite adducts of adenylylsulfate reductase, flavocytochrome b2, pyranose 2-oxidase, cholesterol oxidase, and alditol oxidase have been reported (pdb_00001jnz,^22^ pdb_00001ltd,^23^ pdb_00003lsm,^24^ pdb_00004u2l,^25^ pdb_00002vfv^26^). The sulfite adducts of flavocytochrome b2 and alditol oxidase were obtained by soaking the crystals in Na_2_SO_3_. In the other examples, the sulfite ion resulted from the oxidation of dithionite, which had been used for crystal soaking. In this group of structures, the angle between the N5–S bond vector and the N5–N10 axis is 114° ± 7°. The angle in the highest resolution structure is 120° (pdb_00004u2l, 1.34 Å resolution). The SO_3_^–^ group lies on the *re* or *si* face of the isoalloxazine, which presumably indicates the face available for the reaction with substrates. In all these structures, the isoalloxazine exhibits butterfly bending consistent with two-electron reduction, as shown for cholesterol oxidase (Figure 2B). The bound SO_3_^–^ group typically forms electrostatic interactions with basic and hydrogen-bond-donor side chains.

The sulfite adduct is relevant to the mechanism of adenylylsulfate reductase, which catalyzes the interconversion of adenosine 5′-phosphosulfate and AMP. Fritz et al. captured the sulfite adduct of adenosine 5′-phosphosulfate reductase by soaking crystals of the reduced enzyme with adenosine 5′-phosphosulfate (pdb_00001jnz).^22^ The structure is consistent with a catalytic mechanism that begins with the nucleophilic attack of the N5 atom of reduced FAD on the sulfur atom of adenosine 5′-phosphosulfate, producing the sulfite adduct and AMP. Release of sulfite completes the reaction cycle, consistent with the Sulfite adduct being a reversible covalent modification of flavins.

## Monoamine Oxidase

### Structure and Function of Monoamine Oxidase (MAO)

MAOs are representative of flavoprotein amine oxidases, which make up a large group of enzymes that catalyze the oxidation of C–N bonds. As described by Fitzpatrick,^1^ flavoprotein amine oxidases may be organized into two families based on structural similarity to the archetypes MAO and d-amino acid oxidase. The MAO structural family includes MAO-A/B, l-amino acid oxidases, the KDM1 class of protein lysine demethylases, spermine oxidase, and polyamine oxidase. The d-amino acid oxidase structural family includes the eponymous enzyme and those that oxidize glycine or *N*-methylated amino acids, including sarcosine oxidase.

MAO-A and MAO-B are prototypical flavoenzymes whose chemistry, biological significance, and pharmacology make them central to any review of covalent inactivation mechanisms.^27–29^ These enzymes, located on the outer mitochondrial membrane, catalyze the oxidation of amines to the corresponding imine (Scheme 2). The imine product is hydrolyzed nonenzymatically to the respective aldehyde or ketone.^27^ Physiological substrates include neurotransmitters such as dopamine and serotonin, as well as trace amines, including phenethylamine and tyramine. Following oxidation of the substrate, the reduced flavin is oxidized by O_2_, with formation of H_2_O_2_, a major redox metabolite. Through this activity, MAOs regulate synaptic monoamine levels and modulate the intracellular redox status.

**Scheme 2:**
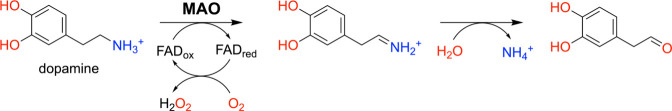
Reaction Catalyzed by MAO, as Illustrated with the Substrate Dopamine

Dysregulated MAO activity has been implicated in neurodegeneration, depression, anxiety disorders, neuroinflammation, and cardiac pathophysiology, underscoring the medical relevance of understanding the structure and mechanism of MAO.^30,31^ MAOs are also gaining attention as potential anticancer targets.^32^ Importantly for this review, MAO-B is among the best-characterized human enzymes known to undergo covalent modification of FAD by clinically relevant mechanism-based inhibitors, making it a cornerstone example of flavoenzyme inactivation.

The first high-resolution structure of human MAO-B revealed an enzyme anchored to the outer mitochondrial membrane via a C-terminal transmembrane helix, existing as a homodimer with each protomer containing a deeply buried 8α-S-cysteinyl-FAD (pdb_00001gos).^33^ MAO exhibits a 3-domain fold (based on analysis by CATH^34^) comprising a 3-layer ββα-sandwich FAD-binding domain, an α-β complex architecture domain, and an α-helical orthogonal bundle membrane association domain (Figure 3A). The isoalloxazine ring of FAD sits at the base of a hydrophobic cavity at the interface between the α-β and membrane association domains.

**Figure 3:**
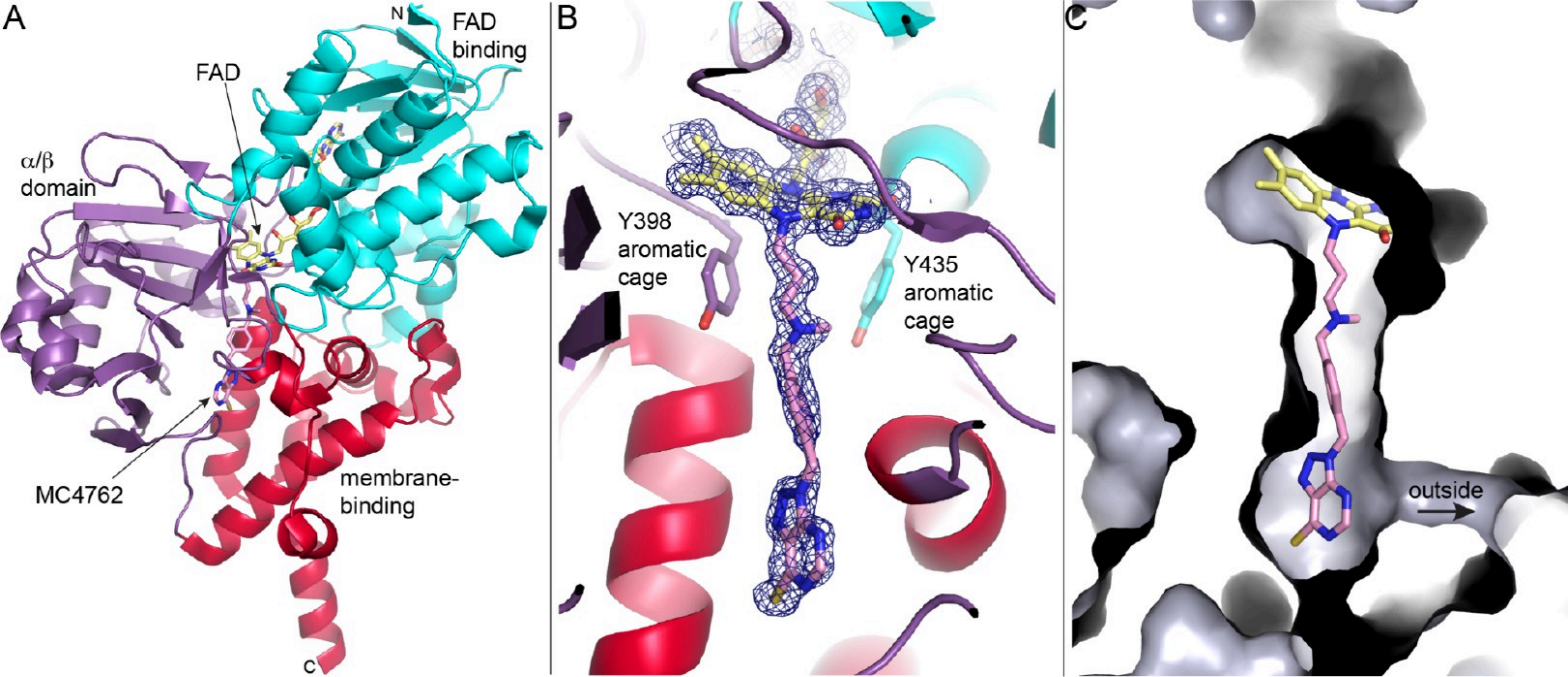
Structure of MAO-B inactivated by propargylamine inhibitor MC4762 (pdb_00009fjt). (A) Fold of MAO-B. The protein is colored by domains. (B) Structure of the covalent adduct resulting from MC4762. The mesh represents a 2*F*_0_-*F*_c_ map (1σ). (C) Surface representation highlighting the ∼17 Å tunnel encasing the adduct.

The hydrophobic cavity of MAO-B binds substrates/inhibitors. The geometry of the hydrophobic cavity is dominated by an aromatic cage comprising two tyrosine residues that contact the *re* face of the isoalloxazine (Tyr398, Tyr435) (Figure 3B). This cage plays multiple roles: it helps orient substrates for oxidation, stabilizes the developing iminium cation in the reductive half-reaction, and constrains the positioning of covalent inhibitors in ways that strongly influence adduct formation and selectivity.^35,36^

The aromatic cage is pivotal for binding substrates and covalent inhibitors. Mutations at Tyr435 compromise efficient alignment of substrates with the FAD *re* face, reduce turnover rates, and decrease the effectiveness of mechanism-based inhibitors.^35^ These findings indicate that covalent inactivation depends not only on the chemistry of the inhibitor but also on precise geometric preorganization enforced by the protein scaffold. Thus, MAO exemplifies the principle that covalent adduct formation arises from an interplay between the flavin reactivity and active-site architecture.

The hydrophobic cavity in MAO-B consists of two chambers (often described as “bipartite”), a substrate cavity, and an entrance cavity. The entrance cavity is separated from the substrate cavity by Ile199. Mutagenesis studies demonstrate that Ile199 functions as a dynamic gate. Mutations that reduce its steric bulk merge the two cavities, altering ligand binding modes and influencing both reversible inhibition and the approach trajectory of mechanism-based inhibitors.^37,38^ These structural insights help explain why MAO-B is highly selective for certain irreversible inhibitors whose aromatic or bulky substituents project into the entrance cavity. Covalent modification of the FAD with large inhibitors, such as the propargylamine inhibitor MC4762, can cause the substrate and entrance cavities to coalesce into a long tunnel, which in the case of MC4762 spans 17 Å (Figure 3C).

The structure of MAO-A was first solved using the rat enzyme inactivated by clorgyline (pdb_00001o5w)^39^ and later with the human enzyme (pdb_00002bxr).^40^ Like MAO-B, MAO-A features an aromatic cage of two tyrosine residues (Tyr407, Tyr444). MAO-A differs from MAO-B in having a single elongated active-site cavity, contrasting with the bipartite cavity of MAO-B. This difference contributes to distinct substrate preferences and inhibitor selectivity between the two isoforms.^33,38^ MAO-A has more polar features in the cavity and lacks the pronounced entrance–substrate division seen in MAO-B. Such differences mean that even when MAO-A and MAO-B form the same type of flavin adduct, the surrounding protein environment stabilizes the adduct differently, leading to variations in spectral signatures, kinetics, and inhibitor potency.

### Catalytic Mechanism of MAO and Chemical Basis for Covalent Trapping

The catalytic mechanism of MAO has been debated. Various mechanisms have been proposed, including single-electron transfer, hydrogen atom transfer, nucleophilic mechanisms, and direct hydride transfer.^1,41^ Fitzpatrick makes a convincing argument, motivated in part by conservation of mechanism across the flavoprotein amine oxidase family, that MAO catalysis likely proceeds through direct hydride transfer from the substrate to the FAD N5, generating reduced flavin and an iminium ion.^42^ Analogously, the oxidation of inhibitors to reactive iminium species is often the first step in the mechanism-based inactivation of MAO and other flavoenzymes. The aromatic cage predisposes certain inhibitor-derived intermediates to react with flavin instead of dissociating. MAO thus provides an environment that is chemically prone to covalent modification.

Two electrophilic centers on the flavin—N5 and C4a—are exploited depending on the inhibitor scaffold and intermediate structure. N5 alkylation predominates for propargylamines, allylamines, and hydrazines, whereas C4a alkylation is observed with cyclopropylamines.

### N5 Alkylation of MAO by Propargylamines

Propargylamine-based inhibitors (Scheme 3) form N5 covalent adducts, following MAO-catalyzed oxidation. Crystal structures of MAO-B inactivated by several propargylamine inhibitors are available, including pargyline,^33^ *N*-propargylmethamphetamine (selegiline, deprenyl),^40^ rasagiline and rasagiline analogs,^43,44^ 1-propargyl-4-styrylpiperidine-like analogues,^45^ F2MPA,^46^ ASS234,^47^ and most recently, MC4762.^48^ The structure of human MAO-A inactivated by clorgyline has also been solved.^40^

**Scheme 3:**
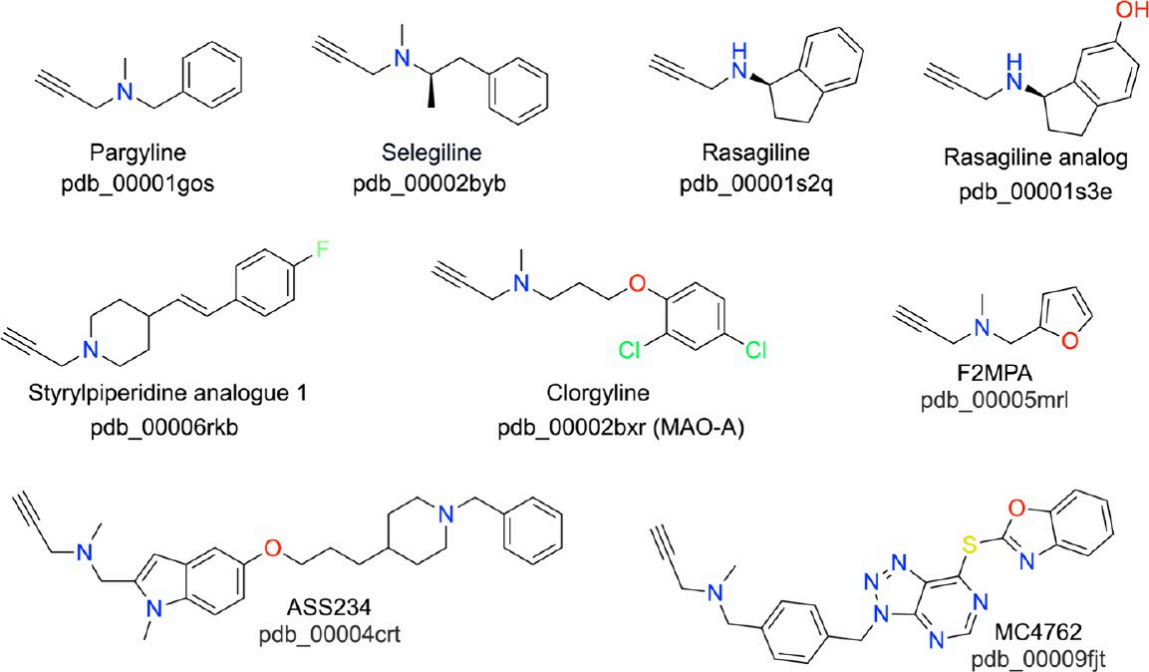
Selected Propargylamine Inhibitors of MAO-B

The structures of MAO inactivated by propargylamines provide insight into the mechanism of inactivation and information relevant to inhibitor development. Primarily, they reveal the site of covalent attachment and the structure of the adduct, i.e., the final product of the inactivation process. This structural information, combined with biochemical, kinetic, and spectroscopic data, enables the proposal of a mechanism of inactivation. Further, the structures show how the aromatic portion of the inactivator engages the active site, which provides insight into the molecular recognition of the inhibitor and how the active site stabilizes the structure of the adduct. Such information is useful for inhibitor design. Also, propargylamine inhibitors of various lengths can serve as molecular rulers used to measure the size and shape of the hydrophobic cavity.

The likely mechanism of propargylamines begins with the enzyme-catalyzed oxidation of the propargylamine to an iminium cation along with a 2-electron reduction of FAD (Scheme 4). Nucleophilic attack by N5 of the reduced FAD on the alkyne moiety of the iminium cation generates a covalent FAD-allenic intermediate (Scheme 4, top). Alternatively, the same intermediate could be formed by attack of N5 on the allenic cation resonance contributor (Scheme 4, bottom).^49^ In either route, water-assisted proton transfer from N5 to the allene results in a stable N5-alkyl-FAD adduct. High-resolution MAO-B crystal structures consistently show an ordered water molecule hydrogen bonding to FAD N5 and O4 that may play this role (e.g., pdb_00002v5z). The positive charge of the covalent adduct is stabilized by resonance delocalization. A more elaborate mechanism featuring C4a adduct intermediates has been proposed from quantum chemistry calculations.^50^

**Scheme 4:**
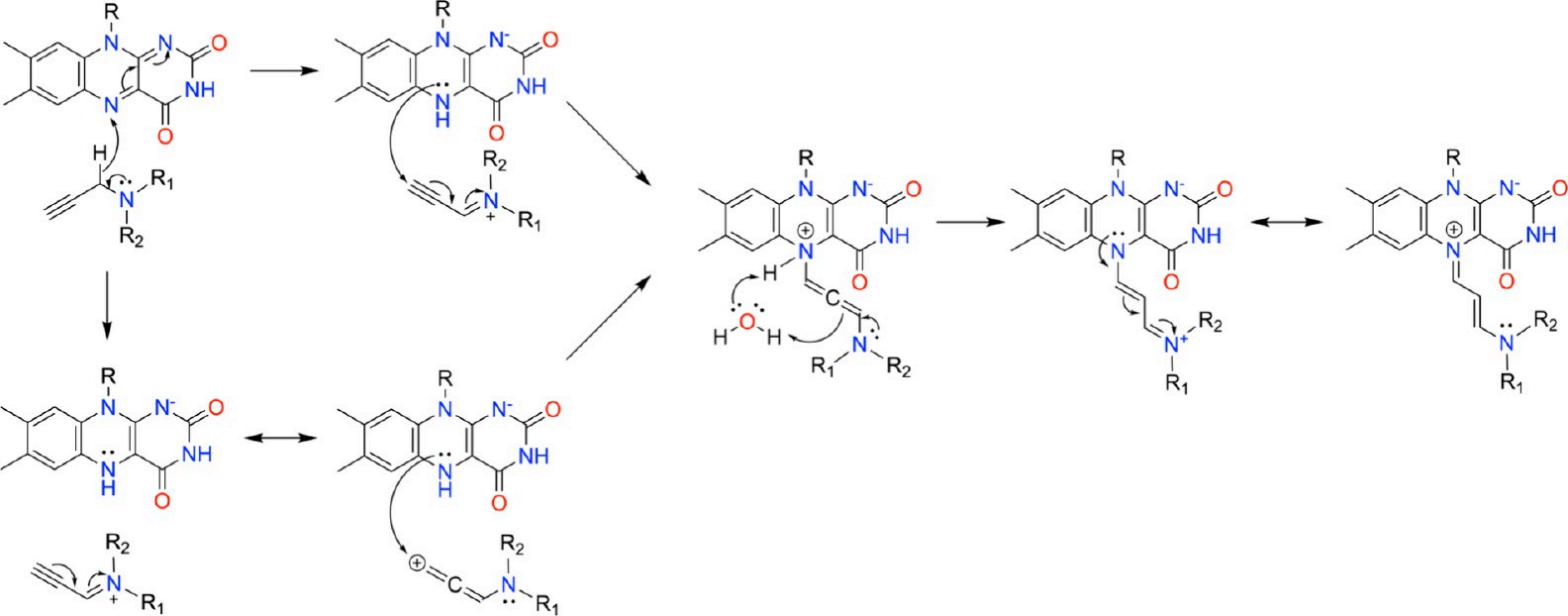
Mechanism of Inactivation of MAO-B by Propargylamine Inhibitors

### N5 Alkylation of MAO by Arylalkylhydrazines

Arylalkylhydrazines also result in covalent modification of N5, but via a mechanism different than that of propargylamines. The crystal structures of MAO-B inactivated by benzylhydrazine and phenyethylhydrazine help illustrate this chemistry (pdb_00002vrl, pdb_00002vrm).^51^ The structures show that N5 is covalently modified with the apparent loss of N_2_ from the inhibitor (Figure 4A). The aromatic ring of the adduct is sandwiched between the tyrosine residues of the aromatic cage. The isoalloxazine exhibits butterfly bending, consistent with a 2-electron reduction. Thus, these inhibitors lock MAO-B into an N5-alkylated reduced state.

**Figure 4:**
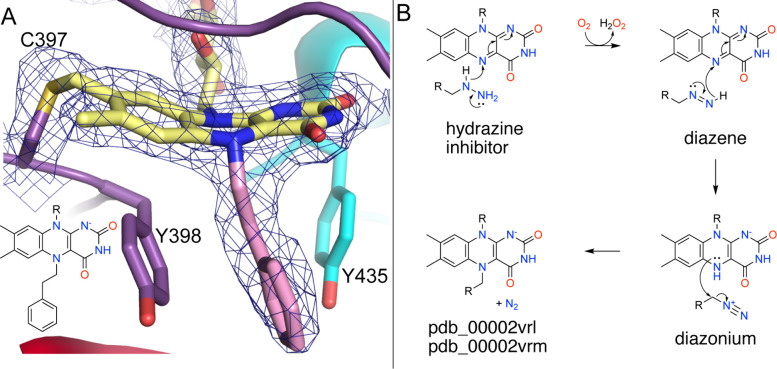
Inactivation of MAO-B by hydrazine inhibitors. (A) Structure of MAO-B inactivated by phenylethylhydrazine (pdb_00002vrm). The mesh represents a 2*F*_0_-*F*_c_ map (1σ). (B) Proposed mechanism of inactivation of MAO-B by arylalkylhydrazines, where R is phenyl or benzyl.

Two mechanisms of inactivation have been proposed based on studies of MAO-B and the related MAO-family enzyme lysine-specific demethylase 1 (LSD1, also known as KDM1A).^51–53^ These mechanisms differ in the nature of the intermediates involved (radical versus diazonium) and in the number of FAD reduction–oxidation cycles required. The diazonium mechanism appears to be more plausible because it involves hydride-transfer steps, and hydride transfer is also likely to occur during substrate oxidation. The requirement for O_2_ and multiple enzyme-catalyzed oxidation steps distinguishes hydrazine-based MAO inactivators from propargylamines and allylamines.

The diazonium mechanism is shown in Figure 4B.^52,53^ Initial oxidation of the inhibitor by hydride transfer generates the diazene and reduced FAD. After reoxidation of the flavin, a second enzyme-catalyzed oxidation yields a primary diazonium species and a two-electron-reduced flavin. Nucleophilic attack by N5 of the reduced flavin, likely accompanied by proton transfer from N5 to water, leads to the formation of the covalent FAD adduct with loss of N_2_.

### N5 Alkylation of MAO by Allylamines

Allylamine-based compounds were some of the first mechanism-based inhibitors of MAO-B to be studied. Early research entertained two mechanisms for the inactivation of MAO by allylamine, one resulting in covalent modification of FAD N5 and another leading to covalent modification of an active-site nucleophilic amino acid residue.^54,55^ The crystal structure of MAO-B inactivated by mofegiline was crucial for clarifying the mechanism of inactivation for this class of inhibitor (pdb_00002vz2).^56^ Mofegiline is a potent covalent inactivator of MAO-B but a weaker reversible inhibitor of MAO-A.^56^ The electron density for mofegiline-inactivated MAO-B showed that FAD N5 was modified (Figure 5A). The electron density was consistent with N5 bonded to an sp^2^ carbon atom and loss of the fluorine atom from the fluoroethene group.

**Figure 5:**
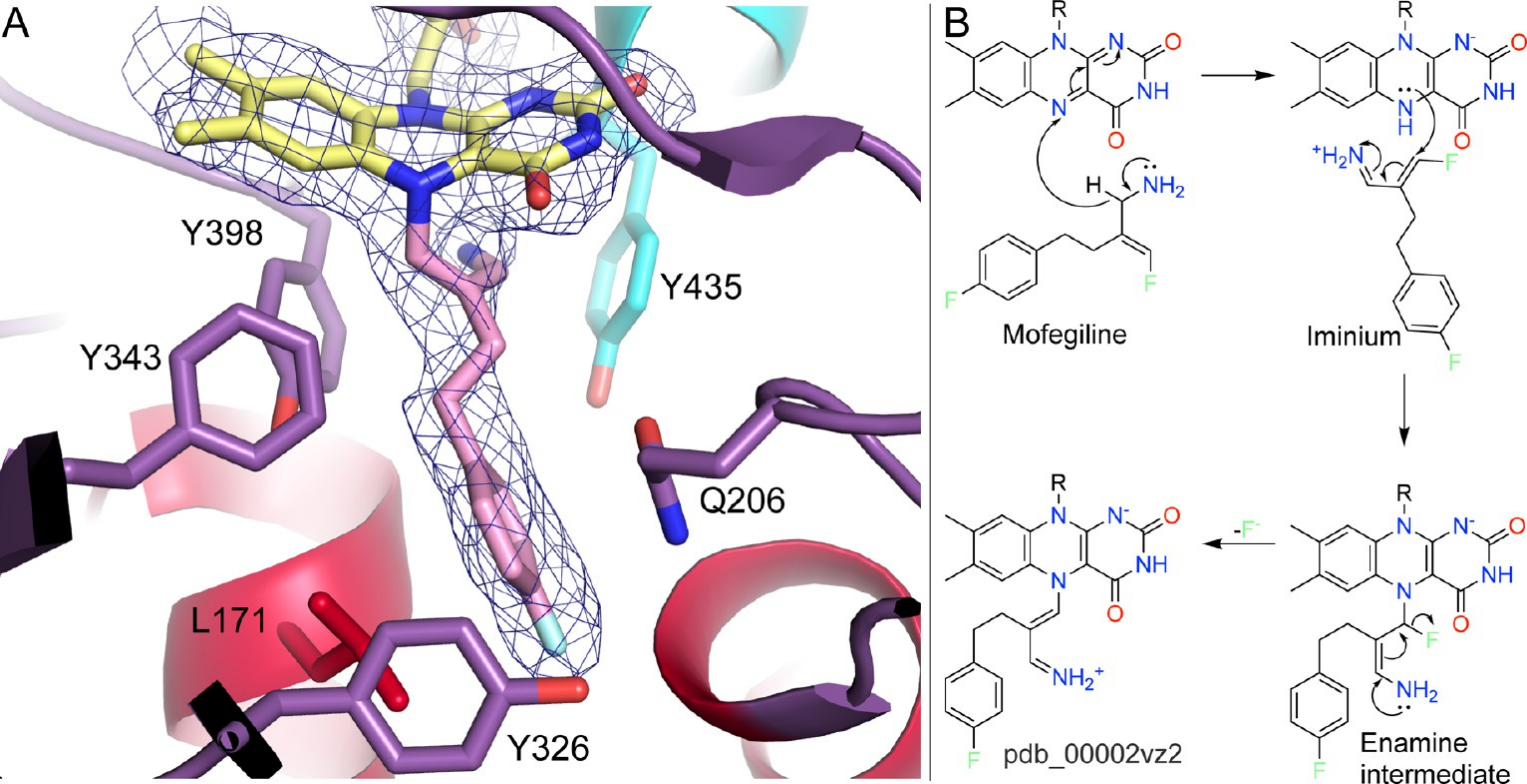
Inactivation of MAO-B by mofegiline. (A) Structure of MAO-B inactivated by mofegiline (pdb_00002vz2). The mesh represents a 2*F*_0_-*F*_c_ map (1σ). (B) Mechanism of mofegiline.

The proposed mechanism for the inactivation of MAO-B by mofegiline^29,56^ begins with oxidation of the inhibitor at the C–N bond, producing an iminium species and reduced flavin (Figure 5B). The iminium intermediate then undergoes Michael addition by N5 of FADH^–^, likely accompanied by proton transfer from N5 to water, to give an enamine intermediate. Because this enamine intermediate is unstable, it eliminates fluoride to form a highly conjugated N5-flavin adduct.

### C4a Alkylation of MAO by Cyclopropylamines

Cyclopropylamine inhibitors follow a mechanistically distinct pathway, resulting in C4a–C flavin adducts. Most of the biochemical studies and all the structural work used *trans*-2-phenylcyclopropylamine (2-PCPA). The mechanism of inactivation of MAO-B by 2-PCPA was debated for several years, beginning in the 1980s. Researchers tended to agree that the mechanism proceeded through one-electron transfer steps and radical intermediates, but disagreed on whether the site of modification was a protein amino acid or the FAD. Interested readers could see Ramsay and Albreht for a history of that debate.^53^

X-ray crystallography helped to clarify the mechanism. Three structures of MAO-B inactivated by 2-PCPA were reported in 2010 (pdb_00002xfu, pdb_00002xcg, pdb_00002xfo).^57^ The latter two structures also contain the noncovalent inhibitor 2-(2-benzofuranyl)-2-imidazoline bound in the entrance of the hydrophobic cavity, situated 14 Å from FAD C4a and 9 Å from the phenyl ring of the inactivator. (Apparently, inactivation by 2-PCPA enhances the binding of this noncovalent inhibitor.) The highest resolution electron density for the C4a modification is found in pdb_00002xcg (1.90 Å). This structure showed that the FAD C4a is connected to the α-carbon (relative to the phenyl group) of 2-PCPA rather than the β-carbon, as had been postulated based on a lower resolution structure (Figure 6A).^58^

**Figure 6:**
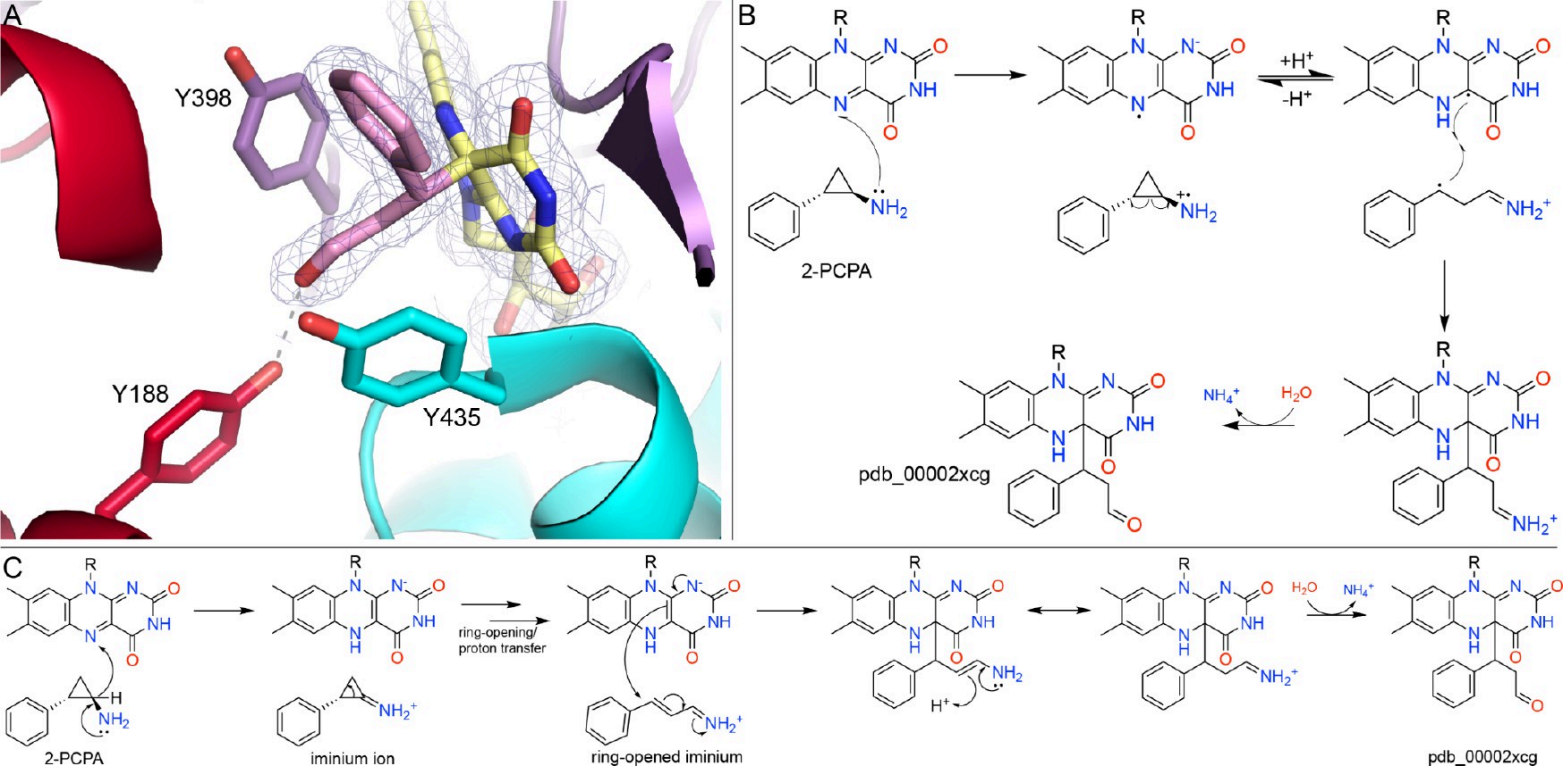
Inactivation of MAO-B by 2-PCPA. (A) Structure of MAO-B inactivated by 2-PCPA (pdb_00002xcg). The mesh represents a 2*F*_0_-*F*_c_ map (1σ). (B) Single-electron transfer mechanism of the inactivation of MAO-B by 2-PCPA. (C) Proposed hydride transfer mechanism of 2-PCPA.

A proposed inactivation mechanism from Silverman^59^ and updated by Schmidt and McCafferty^60^ starts with single-electron transfer from the inhibitor to the oxidized flavin, yielding a flavin semiquinone and a cyclopropylamine radical cation (Figure 6B). Then, the cyclopropyl ring opens, generating a highly reactive benzyl radical iminium cation. The radicals combine to form a C4a-3-phenylpropan-1-iminium adduct. Hydrolysis of the iminium group of the adduct generates the aldehyde adduct modeled in the crystal structure (pdb_00002xcg, Figure 6A).

An alternative mechanism involving hydride transfer is proposed in Figure 6C. 2-PCPA is proposed to undergo flavin-dependent oxidation by hydride transfer to give a cyclopropyl iminium ion intermediate. Ring opening of this species, followed by proton transfer, generates an allylic iminium that can be trapped by the C4a of the reduced flavin to form a covalent C4a adduct. Subsequent hydrolysis gives the adduct observed crystallographically.

### The Suicide Amine Substrate, *N*-(2-aminoethyl)-*p*-chlorobenzamide

The compound *N*-(2-aminoethyl)-*p*-chlorobenzamide is a close analog of lazabemide, a reversible inhibitor of MAO-B that reached clinical trials for Parkinson’s disease in the 1990s.^61^ The absence of an obvious warhead suggests that this compound should behave more like a substrate than a covalent inactivator. Nevertheless, the crystal structure of the inhibited enzyme revealed an N5 adduct with apparent loss of the amino group and incorporation of water (pdb_00001ojc, Figure 7A).^58^ The authors noted “preliminary mechanistic studies” suggesting that the amino group is released prior to forming the N5 adduct.^58^ A possible mechanism that is consistent with this idea begins with the usual oxidation of the inhibitor to an iminium ion, followed by reaction with water to produce an aldehyde and ammonia (Figure 7B). Then, attack of N5 of the reduced FAD on the aldehyde, along with transfer of a proton from the N5 to the carbonyl O atom, yields the adduct derived from the crystal structure.

**Figure 7:**
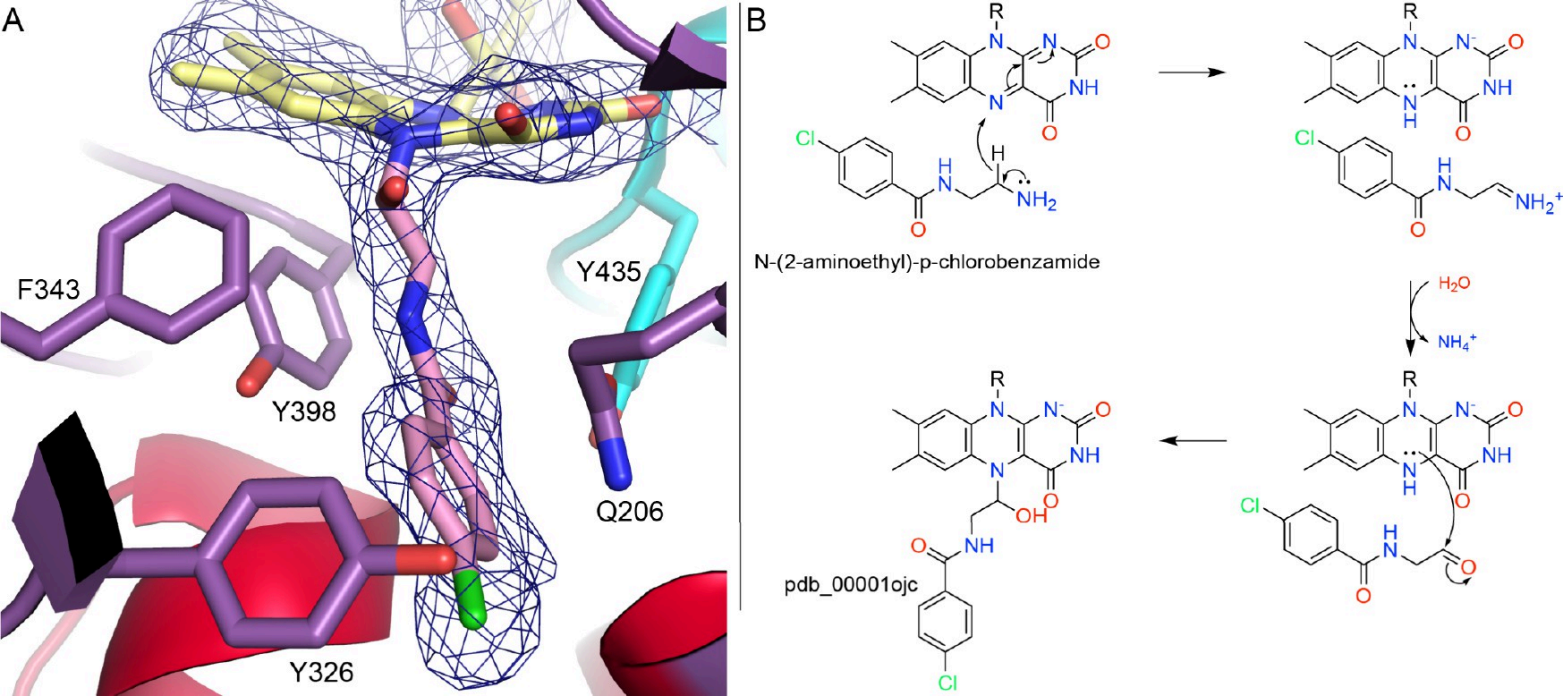
Inactivation of MAO-B by *N*-(2-aminoethyl)-*p*-chlorobenzamide. (A) Structure of the covalent adduct with 2*F*_0_-*F*_c_ (1σ) electron density (pdb_00001ojc). (B) Proposed mechanism of inactivation.

## Lysine-Specific Histone Demethylase 1 (KDM1A/LSD1)

### Structure and Function of LSD1

KDM1-class flavin-dependent protein lysine demethylases are FAD-dependent amine oxidases that catalyze the oxidative demethylation of mono- and dimethylated lysine residues on histone and nonhistone proteins, producing formaldehyde and hydrogen peroxide as byproducts (Scheme 5).^62^ Biologically, KDM1 demethylases act as catalytic subunits within multiprotein chromatin-regulatory complexes, where interactions with coregulators dictate substrate specificity, genomic localization, and transcriptional outcome. Through these complexes, KDM1 enzymes play central roles in epigenetic regulation of gene expression programs governing cellular differentiation, development, and lineage commitment, while their dysregulation contributes to cancer proliferation, metastasis, and viral infection and latency.^62^ Because of these roles, KDM1 demethylases have emerged as potential therapeutic targets, especially KDM1A (a.k.a. LSD1), which has been targeted with covalent inactivators that modify the FAD.

**Scheme 5:**
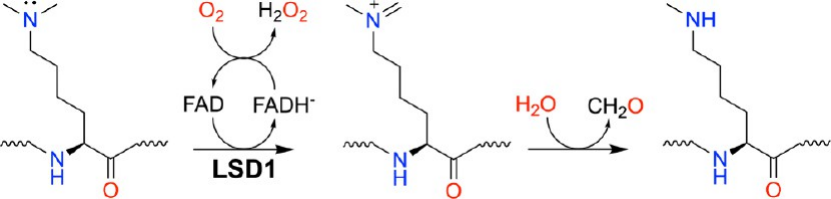
Reaction Catalyzed by LSD1

The catalytic domain of LSD1 adopts the fold of the MAO family of flavoprotein amine oxidases (Figure 8A).^1^ The LSD1 fold also contains an N-terminal SWIRM domain, which is present in several histone-modifying proteins, and a tower domain formed by two long (∼60 and ∼90 Å) α-helices.^63^ The catalytic domain is unique for MAO family enzymes in that its primary structure is interrupted by another domain, i.e., the tower domain. The tower domain serves as a scaffold for binding coregulatory factors, most notably, the corepressor CoREST, forming the catalytically competent LSD1–CoREST core complex. Structural studies have shown that the three-dimensional organization of the active site, particularly the spatial relationship between the FAD isoalloxazine ring, the histone H3 N-terminus, and SNAG-domain transcription factors, plays a decisive role in both the chemical mechanism of covalent inactivation and its biological consequences.^62^

**Figure 8:**
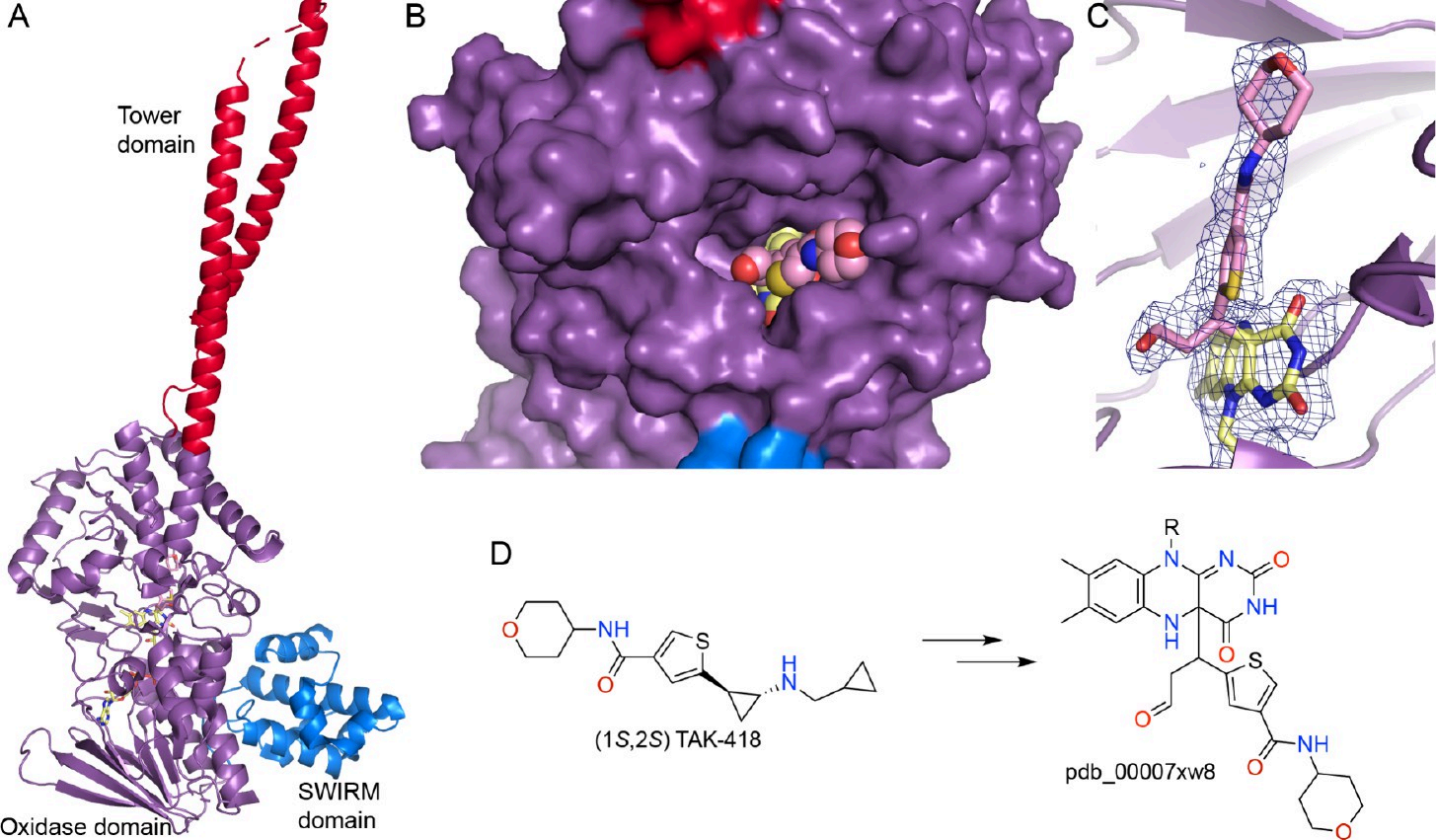
Structure of LSD1 inactivated by (1*S*,2*S*) TAK-418 (pdb_00007xw8). (A) Fold of LSD1. The domains are colored blue (SWIRM), amethyst (oxidase domain), and hot pink (tower). (B) Intrusion of the bulky C4a-linked adduct from (1*S*,2*S*) TAK-418 (spheres) into the SNAG pocket. (C) Electron density for the adduct (2*F*_0_-*F*_c_, 1σ). (D) Chemical structures of TAK-418 and the C4a adduct.

The catalytic pocket of LSD1 is unusually open relative to many chromatin-modifying enzymes, forming a deep but solvent-accessible channel that terminates at the FAD cofactor.^63^ This architecture allows the accommodation of both peptide substrates and relatively bulky small molecules without large backbone rearrangements. Importantly, the substrate-binding channel overlaps with the binding site for SNAG-domain transcription factors, such as GFI1B, which mimic the histone H3 N-terminus. As a result, covalent modification of the flavin has the potential to affect both catalytic activity and protein–protein interactions. A good example is the bulky C4a-linked adduct resulting from the 2-PCPA analog (1*S*,2*S*) TAK-418 (pdb_00007xw8), which intrudes into the SNAG pocket and would presumably inhibit protein–protein interactions (Figure 8B).^64^

### N5 Alkylation of LSD1 by Propargylamine-Derivatized H3 Peptide

The first direct visualization of covalent LSD1 inactivation was achieved by using peptide-tethered suicide substrates. The crystal structure of LSD1-CoREST covalently modified by a propargylamine-derivatized H3 peptide revealed an N5 modification analogous to that observed in MAO-propargylamine complexes (pdb_00002uxn).^65^ The covalent chemistry is consistent with the hydride-transfer/iminium-trapping mechanism outlined for MAO (Scheme 4). This structure simultaneously defined the trajectory of the histone H3 N-terminus and demonstrated that the flavin is positioned to act as an electrophilic trap during mechanism-based inhibition.

### Structural Heterogeneity of Cyclopropylamine-FAD Adducts

The simple cyclopropylamine 2-PCPA was the first covalent inactivator discovered for LSD1,^66^ and cyclopropylamines would become the best-studied class of covalent inactivators. A structure of LSD1-CoREST treated with 2-PCPA provided the first view of small-molecule flavin-directed covalent inhibition of LSD1 (pdb_00002uxx).^67^ In this structure, the inhibitor-derived moiety projects upward from the flavin into the substrate-binding channel, foreshadowing steric overlap with SNAG-domain binding. The structure is notable in the context of the work on MAO-B with 2-PCPA in that the modified FAD features a five-membered cyclic hemiaminal involving the N5 and C4a atoms of the FAD (Figure 9A). Recall that the inactivation of MAO-B by 2-PCPA results in an acyclic C4a modification (Figure 6). As in MAO-B, inactivation proceeds through cyclopropylamine oxidation and ring opening to generate a C4a-linked intermediate. In LSD1, however, this species undergoes reversible intramolecular capture by N5 to form the five-membered cyclic hemiaminal observed crystallographically (Figure 9B).^67^

**Figure 9:**
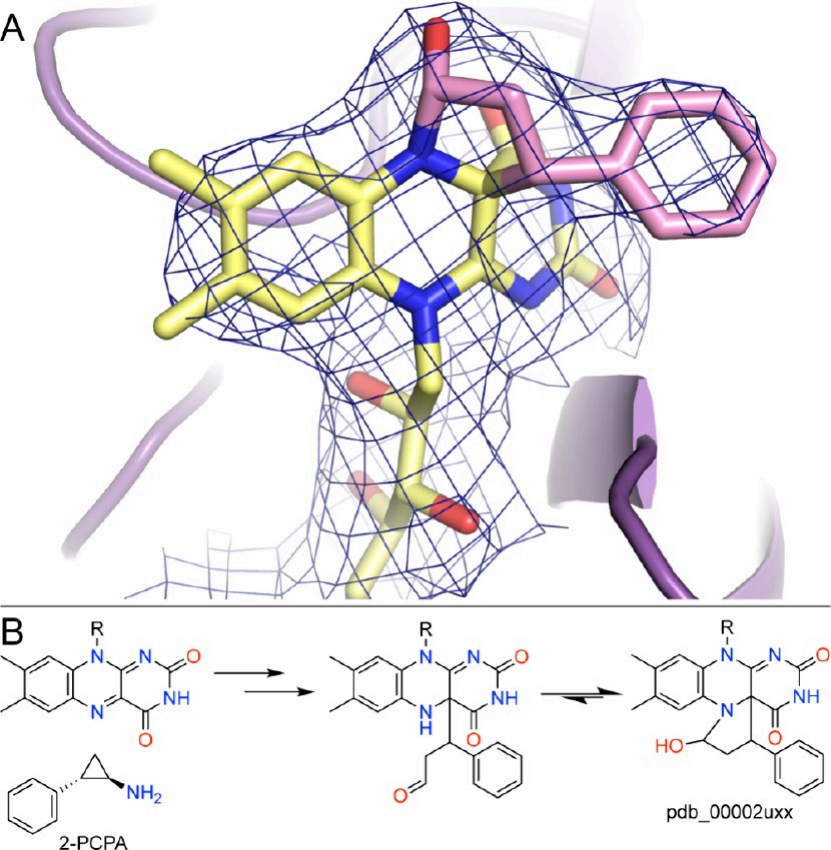
Inactivation of LSD1 by 2-PCPA. (A) Electron density for the cyclic N5–C4a FAD adduct (pdb_00002uxx). The mesh represents a 2*F*_0_-*F*_c_ map (1σ). (B) Proposed mechanism of formation of the cyclic adduct. See the mechanisms in Figure 6 for the omitted steps leading to the C4a adduct.

Higher-resolution and comparative crystallographic analyses clarified that cyclopropylamine-based inhibition does not yield a single immutable adduct geometry. Structures reported by Mimasu et al. captured multiple plausible configurations for the LSD1–2-PCPA adduct, including an N5-linked species, which is distinct from the acyclic C4a adduct characterized in MAO-B. (pdb_00002z3y).^68^ Based on structural and spectral data, the authors suggested that the LSD1–2-PCPA complex is not composed entirely of the five-membered cyclic hemiaminal adduct but contains other species, including the N5 adduct. These data indicated that the flavin isoalloxazine ring of LSD1 can support multiple covalent connectivities without a major rearrangement of the protein scaffold.

Subsequent refinement and comparison with MAO-B structures strengthened this conclusion. Mimasu et al. explored 2-PCPA analogs having larger aromatic groups connected to the cyclopropyl ring.^69^ At the resolution of the structures of ∼3 Å, the authors were not able to distinguish between the five-membered ring model and the N5 model; therefore, the five-membered ring model was used (pdb_00003abt, pdb_00003abu). Collectively, these structures further emphasized partial occupancy and heterogeneity between five-membered ring and N5-linked adduct models and established that LSD1 tolerates a range of flavin adduct geometries while remaining catalytically inactive.

### Structural Mapping of SAR in Early Cyclopropylamine Derivatives

The systematic analysis by Binda et al. helped clarify the relationship between inhibitor structure and the nature of the covalent adduct formed with LSD1.^70^ They performed structural and kinetic analyses of 2-PCPA enantiomers and 2-PCPA analogs in which the phenyl group was replaced by larger substituents having multiple aromatic rings connected by amide bonds. Several structures of inactivated LSD1-CoREST were determined, providing an ensemble view of how substituents are accommodated within the active site (pdb_00002xaf, pdb_00002xag, pdb_00002xah, pdb_00002xaj, pdb_00002xaq, pdb_00002xas).^70^ In these structures, the covalent linkage to FAD N5 is conserved; however, the stereochemistry of the inhibitor dictates the orientation of the installed modification. For example, both the (1*S*,2*R*) and (1*R*,2*S*) enantiomers of 2-PCPA install a 3-phenylpropanal modification, but the linkage to N5 differs, with (1*S*,2*R*) leading to an N5-alkyl modification and (1*R*,2*S*) resulting in N5-acylation (Scheme 6, pdb_00002xah, pdb_00002xaj). Some of the compounds with large aromatic substituents, such as **13b** and **14e**, were found to be ∼200-times better inhibitors than 2-PCPA. These data showed that potency and selectivity could be tuned without altering the fundamental flavin chemistry.

**Scheme 6:**
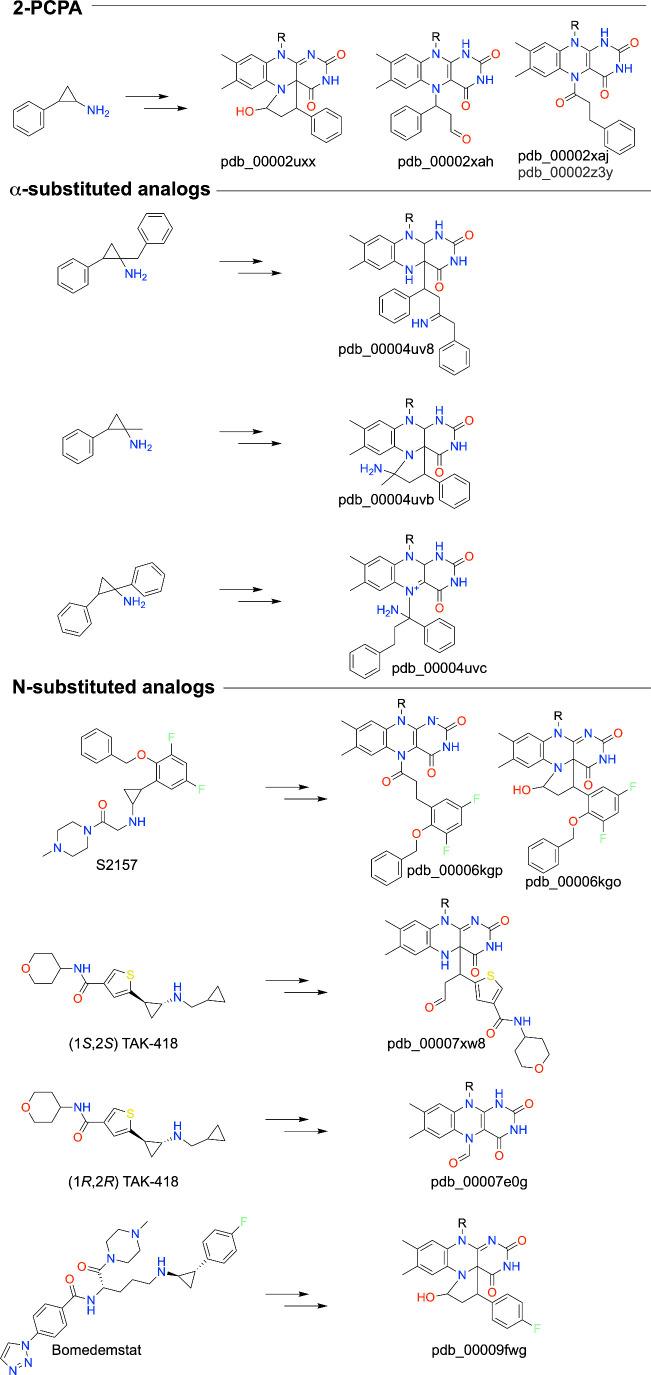
Variety of Covalent Outcomes of 2-PCPA Analogs with LSD1

Rodriguez et al. characterized a similar set of compounds, including some heteroaryl 2-PCPA derivatives that retain covalent flavin modification while improving cellular activity.^71^ Crystallographic confirmation of flavin attachment (N5 adduct) with one of these scaffolds (pdb_00004uxn) reinforced the notion that diverse aromatic systems can be appended without disrupting the core covalent mechanism.

Refinement of cyclopropyl substitution patterns continued with structures reported by Vianello et al., who captured distinct adduct topologies formed by stereoisomeric inhibitors.^72^ The 2-PCPA analogs in this study are unique in that the cyclopropyl ring is α-substituted (with methyl, ethyl, phenyl, and benzyl), which prevents enzyme-catalyzed oxidation of the C–N bond of the inhibitor. The authors propose that ring opening occurs directly, possibly through a radical mechanism. Consistent with Binda et al.,^70^ the stereochemistry dictates the nature of the covalent adduct with FAD. The flavin modifications characterized structurally include three acyclic C4a adducts (pdb_00004uv8, pdb_00004uv9, pdb_00004uva), an acyclic N5 adduct (pdb_00004uvc), and an N5–C4a cyclic species (pdb_00004uvb) (Scheme 6). Altogether, these studies demonstrated that different stereoisomers can form chemically distinct flavin adducts while occupying similar regions of the active site.

### Expansion of Chemical Space to *N*-Substituted Cyclopropylamines

Pivotal structural insight emerged from studies of *N*-alkylated cyclopropylamines. Niwa et al. demonstrated through multiple cocrystal structures that *N*-alkyl substituents are not incorporated into the final flavin adduct (pdb_00006kgk, pdb_00006kgl, pdb_00006kgm, pdb_00006kgn, pdb_00006kgo, pdb_00006kgp, pdb_00006kgq, pdb_00006kgr).^73^ Instead, oxidative ring opening apparently results in the loss of the *N*-substituent, yielding N5-acyl and cyclic N5–C4a hemiaminal FAD modifications. For a given inhibitor, the electron density was consistent with dual occupancy of acyclic and cyclic adducts. These adducts were resolved at 2.25 Å resolution for compound S2157 (pdb_00006kgo, pdb_00006kgp, Scheme 6).[Fn fn1] This work reiterated the theme of structural heterogeneity of LSD1 inactivation by cyclopropylamines and clarified that the cyclopropyl core, rather than peripheral functionality, dictates covalent chemistry. Some of these compounds have inactivation efficiencies over 100 times better than 2-PCPA. The *N*-alkyl substitutions enhanced the potency for LSD1 by 2- to 3-fold. The potency enhancement conferred by the *N*-substituent necessarily reflects the mechanistic steps occurring before adduct formation. The authors suggested that *N*-substituents may enhance the reactivity of the cyclopropyl ring.

The *N*-substituted cyclopropylamine GSK2879552 was useful for understanding the structural basis for separating catalytic inhibition from scaffolding disruption. Cellular studies revealed that many early-generation covalent inhibitors exert biological effects by disrupting LSD1–SNAG-domain interactions rather than by inhibiting demethylation alone.^74^ The authors compared the structures and cellular effects of GSK2879552 with those of the hydrazine class inhibitor phenelzine (2-phenylethylhydrazine). Both inhibitors modify FAD, as expected. The structure of LSD1 inactivated by phenelzine (pdb_00006nr5) showed the N5 adduct observed with MAO-B (pdb_00002vrm), albeit at a modest resolution of 2.9 Å and with atypical geometry. GSK2879552 (pdb_00006nqu) resulted in the N5–C4a cyclic hemiaminal modification observed previously with this class of inhibitor.^73^ Protein structural changes caused by inactivation were analyzed. Phenelzine resulted in structural changes in the α-helical tails of LSD1, which were not observed with GSK2879552. This region mediates CoREST binding, suggesting that phenelzine-induced structural changes in this region may also affect LSD1 activity outside the catalytic region. The authors hypothesized that phenelzine not only inhibits LSD1 catalytic activity but also disrupts LSD1–SNAG-domain interactions, whereas GSK2879552 targets only catalytic activity. They concluded that inhibitors with dual FAD and CoREST-targeting abilities could be important for reprogramming macrophages and potentially initiate an antitumor M1-like phenotype in triple-negative breast cancer and other cancers. However, such “dual” LSD1 inhibitors can cause hematological toxicities that limit dosing.^64,75^

### Fragmentation of *N*-substituted Cyclopropylamine Inhibitors

A new chapter of cyclopropylamine inhibitor research was launched from unexpected results with TAK-418, an *N*-substituted 2-PCPA inhibitor. Structural analysis revealed that TAK-418 forms a tiny FAD adduct consisting of a formyl group attached to N5 (pdb_00007e0g, Scheme 6).^76^ Remarkably, over 90% of the mass of TAK-418 was lost during inactivation in a process known as “fragmentation.” Importantly, this small adduct preserves the geometry of the SNAG-binding channel, allowing for transcription factor binding while fully suppressing catalytic turnover. With this inhibitor, the authors were able to isolate the biological functions of LSD1 enzyme activity without interference from signals derived from LSD1 protein–protein interactions.

Further refinement showed that stereochemistry governs whether fragmentation occurs. Hattori et al. demonstrated that the (1*R*,2*R*) stereoisomer of TAK-418 yields a compact formyl-FAD species, whereas the (1*S*,2*S*) stereoisomer forms a bulky C4a-linked adduct that intrudes into the SNAG pocket (pdb_00007xw8) (Figure 8).^64^ These structures directly linked adduct topology to the biological outcome.

### Mechanism of Fragmentation of *N*-substituted Cyclopropylamine Inhibitors

Although Hattori et al. established a role for stereochemistry in the fragmentation of *N*-substituted 2-PCPA inhibitors, the mechanism of the fragmentation remained unknown. Waterbury et al. addressed this question by capturing multiple stages of inhibitor-FAD adduct formation and fragmentation across an extensive crystallographic series.^75^ The authors developed structure–fragmentation relationships by studying compound T-448, which, like TAK-418, fragments into the N5-formyl adduct, as well as compounds not susceptible to fragmentation. Several FAD covalent adducts from analogs and pure enantiomers of T-448 were obtained by varying the crystal soaking times and conditions. These structures include the hemiaminal ring obtained from a short soak with T-448 (pdb_00008f6s) and an N5-acyl adduct from a longer soak with a nonfragmentable analog (pdb_00008frq). Long soaks with enantiomerically pure T-448 captured a C4a adduct with the less active (1*R*,2*S*) enantiomer (pdb_00009el7) and the hemiaminal ring with the more active (1*S*,2*R*) enantiomer (pdb_00009el8).

Analysis of structural and mass spectral data led to the proposal that fragmentation occurs via a Grob fragmentation mechanism, yielding the N5-formyl adduct and styrene byproducts. The proposed mechanism of fragmentation for T-448 starts with the formation of the cyclic hemiaminal (Scheme 7). Ring opening produces a stable C4a adduct observed with specific stereoisomers of fragmentable inhibitors, including T-448 (pdb_00009el7) and TAK-418 (pdb_00007xw8). Alternatively, ring opening can lead to a carbocation species that has two potential fates, a 1,3-hydride shift into an N5-acyl adduct or fragmentation to the N5-formyl FAD and a styrene molecule. An aryl amide substituent *meta* to the cyclopropyl ring was found to be a key determinant of fragmentation. The ability to visualize both pre- and postfragmentation states established that covalent inhibition of LSD1 by *N*-substituted cyclopropylamines is a dynamic, multistep structural process.

**Scheme 7:**
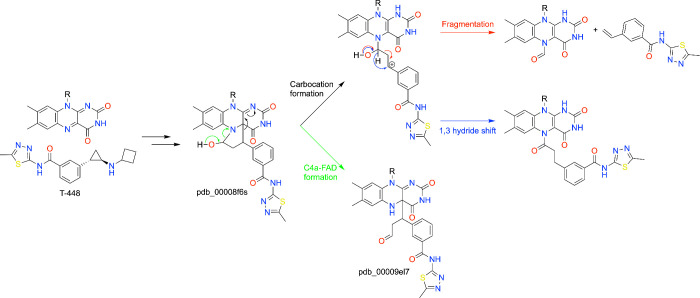
Proposed Mechanism of Fragmentation of *N*-Substituted Cyclopropylamine Inhibitors^a^

a Green, black, blue, and red arrows correspond to C4a-FAD formation, carbocation formation, a 1,3-hydride shift to the N5-acyl adduct, and fragmentation to formyl-FAD, respectively.

### Structural Optimization for Drug-Like Properties and Clinical-Stage Inhibitors

Later medicinal chemistry efforts optimized both pharmacokinetics and safety of 2-PCPA analogs while preserving the desired covalent mechanism. Koda et al. reported structures of optimized S2157 derivatives showing altered adduct connectivity (without fragmentation) and improved microsomal stability (pdb_00007vqs, pdb_00007vqt, pdb_00007vqu).^77^ Parallel work expanded the analysis to cis/trans 2-PCPA derivatives and LSD2 complexes, providing a structural data set for predicting selectivity (pdb_00007w3l, pdb_00007xe1, pdb_00007xe2, pdb_00007xe3).^78^ The 2-PCPA derivative Bomedemstat was structurally characterized as an irreversible LSD1 inhibitor forming a covalent flavin N5–C4a cyclic adduct (Scheme 6, pdb_00009fwg).^79^ CNS-penetrant inhibitors such as S2172 form a classical cyclopropylamine-derived N5 adduct while achieving brain exposure (pdb_00008inl), demonstrating that covalent LSD1 inhibition is compatible with central nervous system drug design.^80^

## Proline Dehydrogenase (PRODH)

### Structure and Function of PRODH

PRODH is the first enzyme of the proline catabolic pathway and catalyzes the flavin-dependent oxidation of l-proline to Δ^1^-pyrroline-5-carboxylate (P5C) (Scheme 8).^81^ In humans, the related enzyme HYPDH (a.k.a. PRODH2, ∼40% identical to PRODH) functions in hydroxyproline catabolism by catalyzing the oxidation of trans-4-hydroxy-l-proline. Both human PRODH and HYPDH are localized to the inner mitochondrial membrane. In some bacteria, PRODH is combined with the second enzyme of the pathway, l-glutamate-γ-semialdehyde dehydrogenase (GSALDH, a.k.a. ALDH4A1), into the bifunctional enzyme known as proline utilization A (PutA).^82^ ALDH4A1 catalyzes the NAD^+^-dependent oxidation of l-glutamate-γ-semialdehyde to l-glutamate (Scheme 8). Historically, bacterial PRODHs and PutAs have been used for kinetic and structural studies because human PRODH/HYPDH enzymes have not been amenable to large-scale recombinant protein expression and structural biology. Nevertheless, several studies have shown that the noncovalent inhibitors and covalent inactivators developed using bacterial PRODHs and PutAs show activity in human cells and animal disease models.^83–90^

**Scheme 8:**
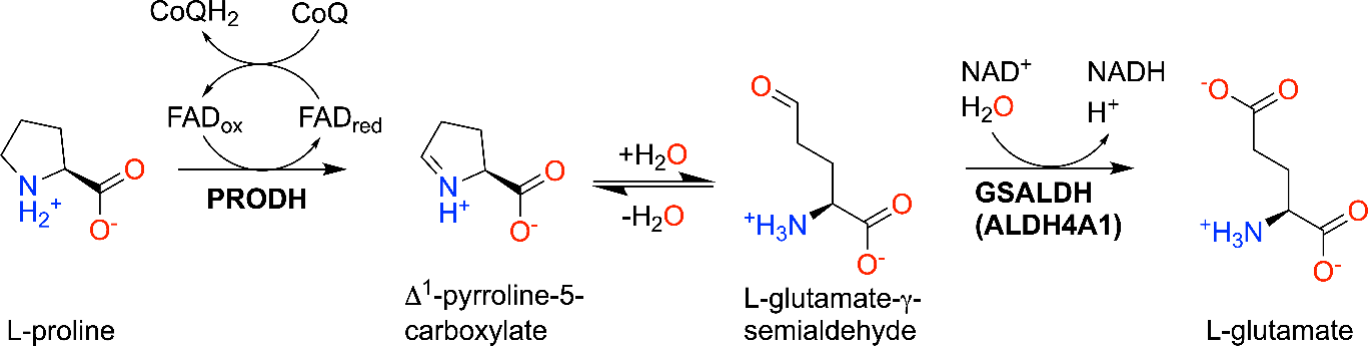
Reactions of Proline Catabolism

X-ray crystallography revealed the fold and active site architecture of PRODH.^81^ The first structure of PRODH was determined with an engineered protein construct containing the PRODH domain of *Escherichia coli* PutA.^91^ This construct has been useful for characterizing both noncovalent^92,93^ and covalent^94^ inhibitors. Similarly, a PRODH domain construct of PutA from *Sinorhizobium meliloti* has been used for the high-resolution structural characterization of PRODH-inhibitor complexes.^95^ Several structures of full-length PutAs have also been determined.^96–100^ Certain bacteria lack *putA* genes and instead produce PRODH and ALDH4A1 as separate enzymes encoded by distinct genes; the structures of the monofunctional PRODHs from *Thermus thermophilus* ^101,102^ and *Deinococcus radiodurans* ^103^ have been determined.

PRODH adopts a variation of the classic (βα)_8_ barrel (TIM barrel) fold, as seen in the structure of the monofunctional PRODH from *T. thermophilus* (Figure 10A). The flavin is bound at the C-termini of the strands of the barrel, which is the canonical active site location for (βα)_8_ flavoproteins. PutAs utilizes FAD, whereas some bacterial monofunctional enzymes can use either FAD or FMN as cofactor.^102^ The human enzymes are thought to use FAD. In PutA, the PRODH barrel is one of five domains (Figure 10B). Three of these domains—arm, alpha, and ALDHSF—are noncatalytic and are thought to be important for maintaining a consistent distance between the PRODH and GSALDH active sites of 42 Å and formation of the substrate channeling tunnel. Structures of human PRODH and HYPDH have not been determined; however, the AlphaFold models suggest that they adopt the core (βα)_8_ barrel fold as the bacterial enzymes. Interestingly, the human PRODH/HYPDH models resemble the elaborated PRODH domains of PutA more than the minimalist fold of bacterial monofunctional PRODHs.

**Figure 10:**
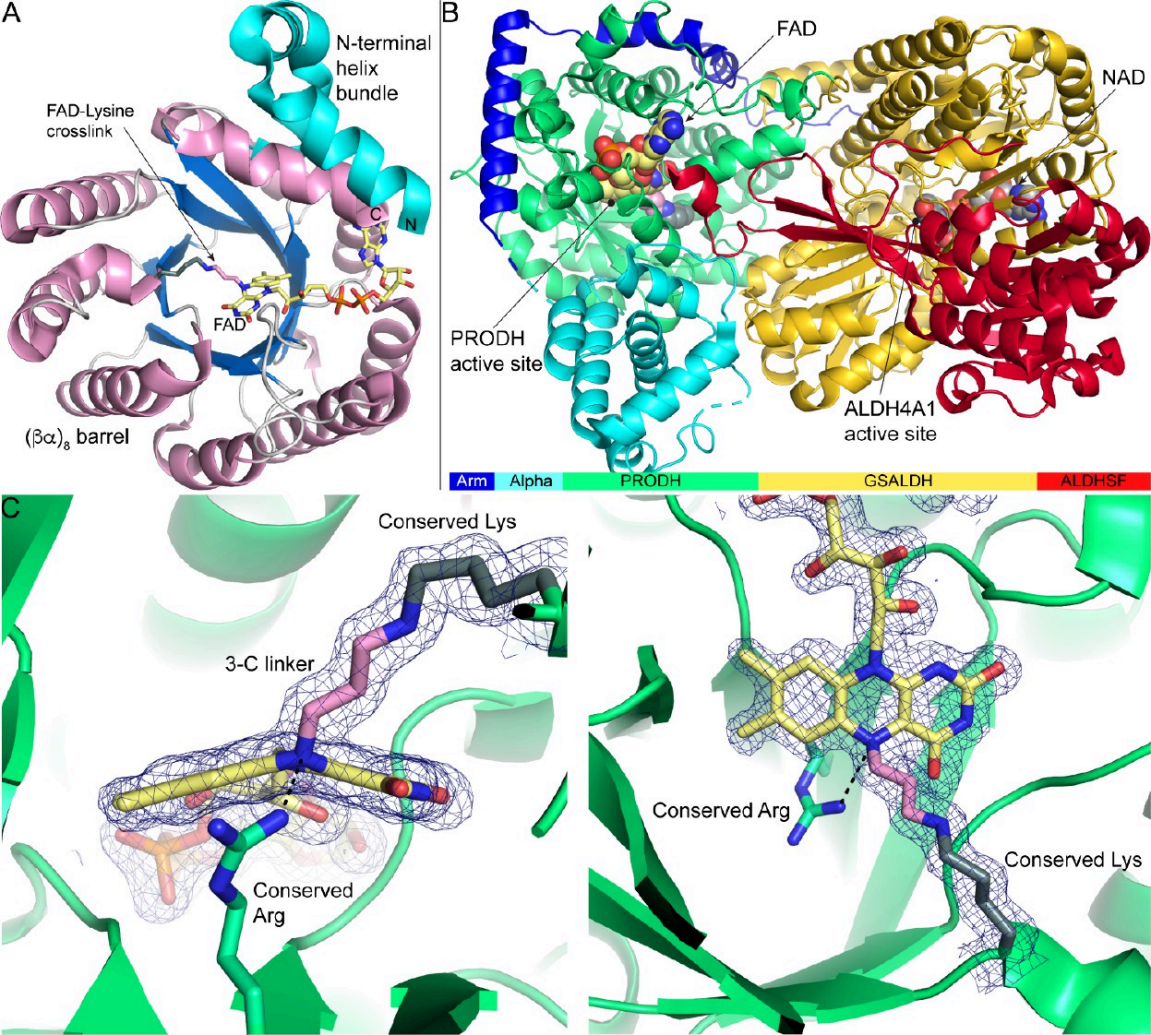
Structure of PRODH and proline utilization A (PutA) inactivated by *N*-propargylglycine. (A) Monofunctional PRODH from *Thermus thermophilus* (pdb_00002ekg). The (βα)_8_ barrel is colored according to the secondary structure. The *N*-terminal helix bundle domain is colored in cyan. (B) PutA from *Sinorhizobium meliloti* (pdb_00009e5w). The domains are colored according to the domain diagram. (C) Two views of the electron density (2*F*_0_-*F*_c_, 1σ) for the covalent FAD-lysine cross-link in PutA (pdb_00009e5w). The butterfly angle of the isoalloxazine is 29°.

Many structures of PRODH with noncovalent inhibitors are available. The most relevant one for understanding the catalytic mechanism is the structure of PRODH complexed with the isostructural proline analogue S-(−)-tetrahydro-2-furoic acid (THFA). This structure is a good model of the Michaelis complex of the oxidized enzyme with the substrate proline and is consistent with a hydride transfer mechanism (pdb_00001tiw, pdb_00004h6q, pdb_00005kf6, pdb_00008dkp).^92,95,98,103^ THFA is a competitive inhibitor (with proline) of PRODH and displays activity in cellular and animal models of cancer.^83,90^

### PRODH and HYPDH as Inhibitor Targets

Inhibition of PRODH is of interest in the context of cancer metabolism.^104^ PRODH and the proline biosynthetic enzyme PYCR1 (catalyzes the NAD­(P)­H-dependent reduction of P5C to l-proline) form a metabolic hub known as the proline cycle,^105,106^ which supports ATP production, protein and nucleotide synthesis, anaplerosis, and redox homeostasis in cancer cells.^104^ The proline cycle plays an especially important role in breast cancer metastasis by supporting the cascade of cellular changes leading to metastasis. Human breast metastatic tissue exhibits upregulated expression of PRODH and PYCR1 compared to primary breast tumor tissue.^83^ Mechanistically, the proline cycle, comprising PRODH and PYCR1 activities, allows metastasizing breast cancer cells to produce ATP at the expense of NADPH, fostering metastatic seeding.^83^

HYPDH is a promising target for the development of drugs to treat primary hyperoxaluria.^86,107–109^ HYPDH catalyzes the first step in the catabolism of 4-hydroxyproline, ultimately leading to glyoxylate, a highly reactive aldehyde.^110^ Normally, glyoxylate is rapidly processed to glycine in peroxisomes or glycolate in the mitochondria or cytosol. The remainder is oxidized to oxalate, which must be renally secreted to avoid the buildup of tissue-damaging calcium oxalate crystals. Aberrant glyoxylate catabolism underlies primary hyperoxaluria, a rare but debilitating set of monogenic diseases in which abnormally high oxalate levels lead to calcium oxalate deposition in the kidneys, nephrocalcinosis, and end-stage kidney failure.^108^ The contribution of 4-hydroxyproline catabolism to oxalate production observed in people with primary hyperoxaluria raises the possibility that a therapy blocking HYPDH activity could be efficacious in lowering urinary oxalate in this group.^108^

### *N*-propargylglycine

Motivated by research on the inactivation of MAO-B by propargylamines, *N*-propargylglycine (NPPG) was explored as a mechanism-based inactivator of PRODH.^111^ NPPG consists of a recognition module (glycine) that guides the compound into the active site and an electrophilic warhead (ethyne) that participates in mechanism-based inactivation. The inactivation of monofunctional PRODH, PutA PRODH domains, and full-length PutAs by NPPG has been characterized with time-dependent kinetics, absorbance spectroscopy, and high-resolution X-ray crystallography (pdb_00002ekg, pdb_00003itg, pdb_00004nme, pdb_00004nmf, pdb_00005ur2, pdb_00009e5w).^89,94,95,97,100,111^ The inhibition is time-dependent and irreversible, with spectral features indicating reduction of the FAD and modification of the N5 atom.

High-resolution X-ray crystallography revealed the nature of the modified FAD from NPPG. The modification is unique among propargylamine-inactivated flavoenzymes in that the FAD N5 is cross-linked to the ε-amino group of a lysine residue (Figure 10C). The N5-lysine linker consists of the three carbon atoms derived from the warhead, whereas the glycine recognition module is absent in the final inactivated enzyme. The N5-alkylated FAD of the NPPG-inactivated PRODH is reduced. Evidence consistent with a 2-electron reduction includes the pale-yellow color of crystals of the inactivated enzyme, the decrease of absorbance near 450 nm during inactivation, and the large butterfly angle of the isoalloxazine ring (Figure 10C). The butterfly angle in NPPG-inactivated PRODHs is in the range of 22–32° (average of 28° ± 3°) (*si* face convex).^89,94,95,97,100,111^

Inactivation by NPPG perturbs the active site structure of PRODH. Installation of the FAD-lysine cross-link is accompanied by remodeling of a key active site helix (α8) and rupture of the ion pair gate, resulting in a more open and disordered active site. These distortions of the PRODH conformation are thought to contribute to the induction of the mitochondrial unfolded protein response in cells by NPPG, resulting in a lowering of PRODH and HYPDH levels.^84–86,89^

The active site lysine is conserved in all PRODHs from bacteria to humans, including human HYPDH, implying that the human enzymes should be susceptible to inactivation by NPPG. Indeed, NPPG has proven to be a useful chemical probe for investigating the roles of PRODH in cancer and PRODH2 in hyperoxaluria.^84–86,89^

Two mechanisms of inactivation of PRODH by NPPG have been proposed (Scheme 9). Both begin with the oxidation of NPPG to *N*-propargyliminoglycine with concomitant reduction of the FAD, analogous to the oxidation of the substrate proline to P5C. Note that this step also occurs in the inactivation of MAO by propargylamine inhibitors (Scheme 4). The two NPPG mechanisms diverge at this point. In one, the N5 atom of the reduced FAD attacks the electrophilic ethynyl group of *N*-propargyliminoglycine, resulting in an allenic species that rearranges, possibly through water-assisted proton transfer from the N5 to the allene, into a conjugated iminium (Scheme 9, top). The conserved arginine near the FAD N5 may encourage the proton transfer step by lowering the p*K*_a_ of the N5–H of the reduced FAD from its solution value of ∼20 (Figure 10C).^112^ We note that a hydrogen bond donor to the N5, located on the flavin side opposite to that facing the substrate (as in PRODH), is a recurrent theme of flavin-dependent dehydrogenases.^113^ The ε-amino group of the conserved active site lysine residue can then attack the iminium adduct, which displaces glycine and results in the formation of an imine linkage between the lysine side chain and modified flavin. As noted for MAO with propargylamines, one may think of the initial attack of the N5 as being directed at an allenic cation resonance contributor rather than the ethynyl group (see Scheme 4).

**Scheme 9:**
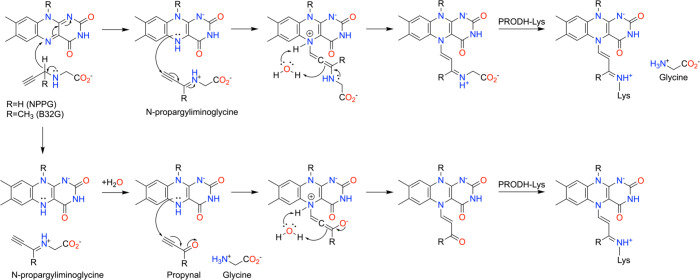
Proposed Mechanisms of Inactivation of PRODH by NPPG

The second proposed mechanism differs in that glycine is jettisoned early in the process (Scheme 9, bottom). Following oxidation of NPPG, *N*-propargyliminoglycine is hydrolyzed to propynal and glycine. The hydrolysis step is analogous to the hydrolysis of P5C to l-glutamate-γ-semialdehyde, which is the substrate for the second enzyme of proline catabolism. Modification of the flavin N5 occurs by the route described above, where propynal is the electrophile. Nucleophilic attack by the N5 of the reduced FAD on the ethynyl group of propynal generates an allenic species that rearranges to an FAD-acrylaldehyde adduct. Attack of the active site lysine on the aldehyde group of the latter results in an imine linkage between the lysine side chain and the FAD N5. In this mechanism, *N*-propargyliminoglycine must undergo hydrolysis either on the enzyme or in solution. Because the hydrolysis of P5C is thought to be a nonenzymatic process, it could be argued that *N*-propargyliminoglycine will also be hydrolyzed in solution. This step, and the necessary rebinding of propynal to the enzyme, make this route the least probable of the two mechanisms.

### Analogs of NPPG

Two close analogs of NPPG have been studied, but-3-yn-2-ylglycine (B32G) and *N*-allylglycine. B32G has a methyl group on the oxidizable C atom of the warhead (Scheme 9). In *N*-allylglycine, the allyl group replaces the alkyne warhead of NPPG.

B32G follows the mechanism of NPPG.^89^ Both isomers of B32G were tested, but only the S-isomer was active. As with NPPG, the crystal structures show that inactivation by S–B32G cross-links the N5 of the FAD to an active site lysine and induces large butterfly bending of the isoalloxazine of ∼30° (pdb_00009d7l).^89^

*N*-allylglycine, the allyl analog of NPPG, installs a different covalent modification than NPPG and follows a different mechanism.^87^ The electron density from crystal structures of the *N*-allylglycine-inactivated enzyme suggested that the N5 was covalently modified by a linear substituent having 4–5 atoms, without the FAD-lysine cross-link (Figure 11A). The modification was ultimately modeled as propanal based on mechanistic reasoning.

**Figure 11:**
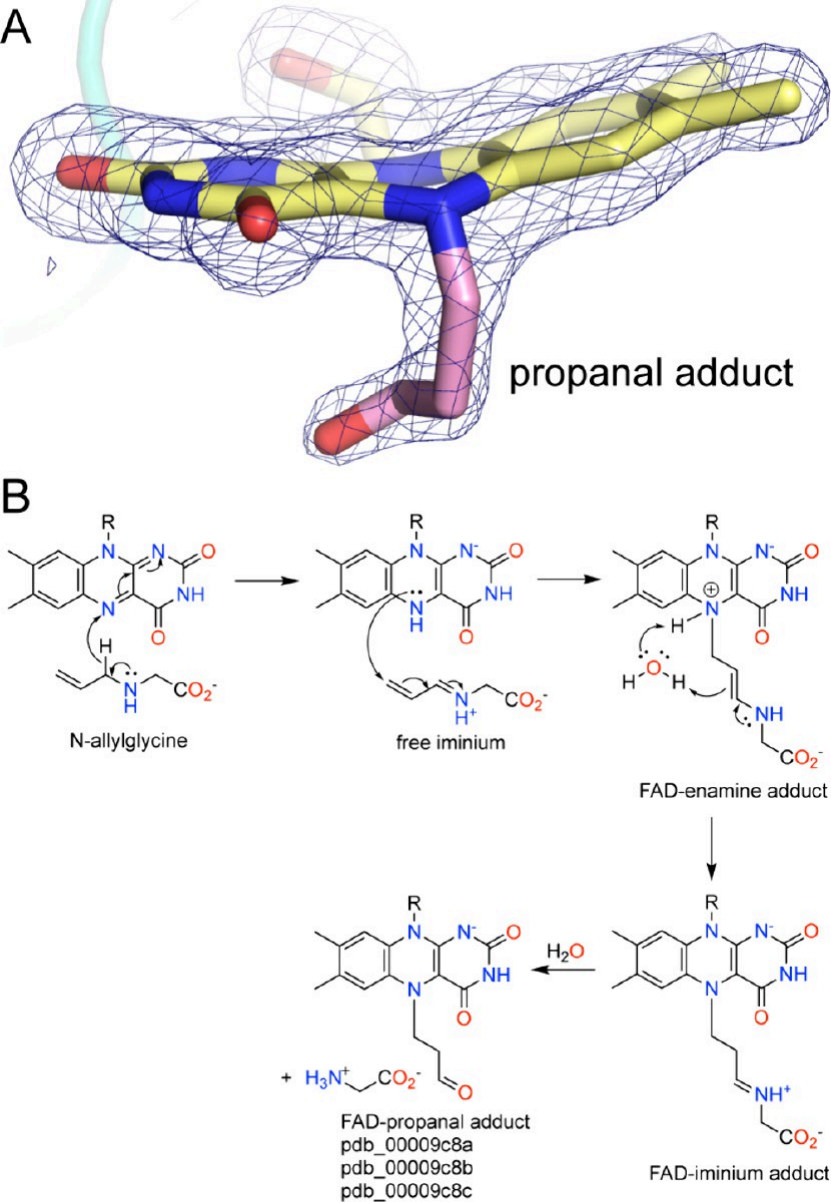
Inactivation of PRODH by *N*-allylglycine. (A) Electron density (2*F*_0_-*F*_c_, 1σ) for the modified FAD in PutA (pdb_00009c8c). The butterfly angle is 18°. (B) Proposed mechanism.

The proposed mechanism of *N*-allylglycine begins with the oxidation of the C–N bond of the inactivator, resulting in an iminium ion and reduced FAD (Figure 11B). Nucleophilic attack by the N5 of the reduced FAD on the allyl group of the iminium ion produces an FAD-enamine covalent adduct. Water-assisted proton transfer from the N5 to the enamine is proposed to generate an FAD-iminium adduct. The electron density observed in the crystal structure of the inactivated enzyme indicated that the adduct was substantially smaller than either the enamine or iminium. This led to the proposal of hydrolysis of the FAD-iminium adduct, releasing glycine and generating an FAD-propanal species. The alkylated FAD of the inactivated enzyme is reduced, based on the characteristic decrease of absorbance near 450 nm during inactivation and the large butterfly bend angle of the isoalloxazine of 17–28° (average of 23° ± 4°) (*si* face convex). Note that the butterfly angle of the N5-propanal isoalloxazine is ∼5° smaller than that of the cross-linked FAD resulting from NPPG.

Like most Hollywood movie franchises, the original NPPG is the best or, in this case, the most efficient. The inactivation efficiencies of NPPG and analogs have been measured using time-dependent inactivation assays, either the classic Kitz and Wilson approach^114^ or a progress curve approach.^115^ These experiments yield estimates of the inactivation parameters *k*_inact_ and *K*_I_. *k*_inact_ is the first-order rate constant describing the maximum potential rate of covalent bond formation (rate at infinite concentration of the inactivator), and *K*_I_ describes the concentration of inhibitor required for half of the maximum potential rate of covalent bond formation.^116,117^ The ratio *k*_inact/_ *K*_I_ is a second-order rate constant describing the efficiency of covalent inactivation. The efficiency of NPPG has been measured for three bacterial PRODHs and is in the range of 0.2–30 M^–1^s^–1^. This variation reflects both the inherent catalytic differences of the enzymes and the details of the activity assays. Comparing results from the same enzyme and assay, the efficiencies of S-B32G and N-allylglycine are 600- and 20-times lower than that of NPPG, respectively.^87,89,95^ The results for S-B32G suggest that a minor modification of the recognition module of NPPG by a methyl group substantially diminished the inactivation efficiency. This likely reflects a steric clash of the methyl group in the highly congested PRODH active site. The lower efficiency of *N*-allylglycine speaks to the importance of the FAD-lysine cross-link for the efficiency of NPPG. In light of these results, improving efficiency by modifying NPPG may be challenging. An alternative strategy, which has not been explored, is to target the active site lysine residue with electrophiles.^118,119^ The fact that this residue functions as a nucleophile in the mechanism of NPPG and S–B32G suggests that it may be susceptible to electrophiles.

### l-thiazolidine-2-Carboxylate (L-T2C)

L-T2C was serendipitously discovered to form an N5 covalent adduct with PRODH.^120^ This molecule emerged during a crystallographic screening campaign of proline analogs against PutA, which eventually yielded several hydroxyproline inhibitors of the PutA ALDH4A1 active site.^121^ Co-crystallization of PutA with L-T2C produced colorless crystals, indicating that the FAD was reduced. The electron density was interpreted as the covalent attachment of L-T2C to the FAD N5 (Figure 12A). The modified isoalloxazine exhibits strong butterfly bending (19–22°) consistent with reduction of FAD. Inhibition of PRODH activity by L-T2C was time-dependent, consistent with covalent inactivation. However, the rate of inactivation was very slow, occurring on a timescale of days.

**Figure 12:**
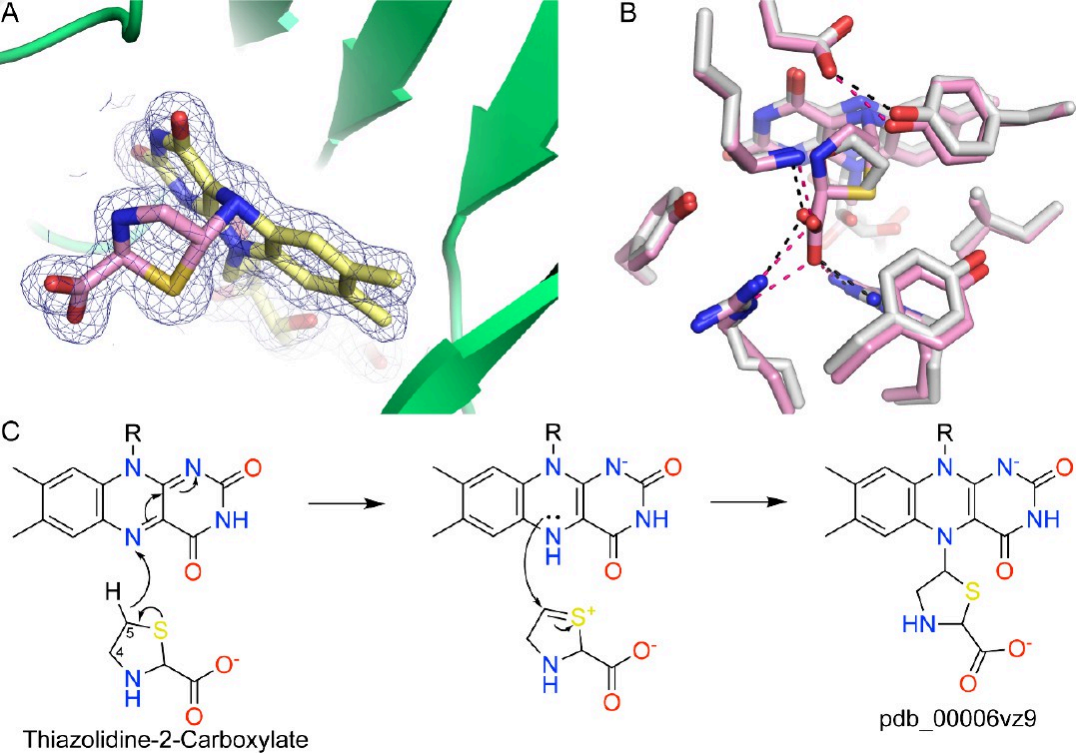
Inactivation of PRODH by L-T2C. (A) Electron density (2*F*_0_-*F*_c_, 1σ) for the modified FAD in PutA (pdb_00006vz9). (B) Comparison of the active sites of PRODH inhibited by L-T2C (pink) and the proline analogue THFA (white). The pink and black dashes represent electrostatic interactions in the T2C and THFA complexes, respectively. (C) Proposed mechanism of inactivation.

The L-T2C-inactivated active site resembles the Michaelis complex, as represented by the structure of PRODH with the proline analogue THFA. Like the Michaelis complex, the active site of the L-T2C-inactivated enzyme is highly ordered, and all the residues that stabilize THFA also contact L-T2C (Figure 12B). This contrasts with the FAD-lysine cross-link installed by NPPG, which severely distorts the active site structure.

A minimal mechanism for L-T2C inactivation^120^ begins with oxidation at C5 of L-T2C, producing a sulfur-stabilized carbocation and the two-electron-reduced FAD (Figure 12C). The sulfur-stabilized carbocation arising from oxidation at C5 may be more reactive than the iminium ion resulting from oxidation at C4.^122^ Nucleophilic attack by the N5 of the reduced FAD at C5 of the sulfur-stabilized carbocation, likely accompanied by proton transfer from N5 to the solvent, yields the covalently modified FAD observed crystallographically. Although the existence of the covalent N5–C5 adduct is well supported, the exact elementary steps of its formation remain uncertain, and the possibility cannot be excluded that adduct formation occurs through a side reaction of an intermediate or product.

### Photoinduced-Covalent Inactivators

This class of PRODH inactivator was serendipitously discovered during follow-up experiments to the L-T2C study.^123^ The two compounds used, 1,3-dithiolane-2-carboxylate (D2C) and tetrahydrothiophene-2-carboxylate (C2C), resemble T2C but lack the amine group to eliminate the possibility of oxidation of the C–N bond, which had been a confounding factor in formulating the mechanism for L-T2C. As expected, cocrystallization of PutA with C2C or D2C yielded yellow crystals, consistent with noncovalent binding to the PRODH active site. Surprisingly, illumination of the crystals with white light from a microscope during crystal harvesting bleached the yellow color, indicating that the FAD had been reduced. The crystal structures showed that the FAD was not only reduced but also covalently modified at the N5 atom by dithiolane (from D2C, pdb_00007mya) or tetrahydrothiophene (from C2C, pdb_00007myc) (Figure 13A).

**Figure 13:**
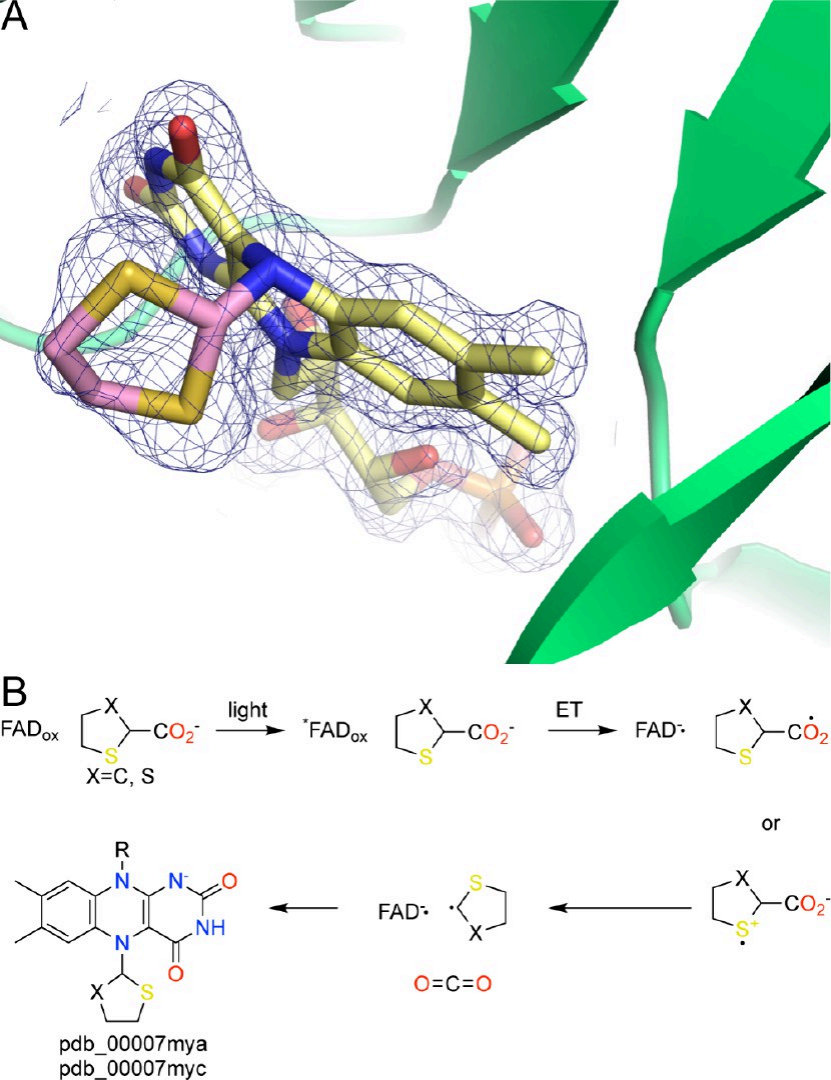
Photoinduced covalent inactivation of PRODH. (A) Electron density (2*F*_0_-*F*_c_, 1σ) for the modified FAD resulting from D2C (pdb_00007mya). (B) Proposed mechanism.

Biochemical experiments provided insight into the mechanism of inactivation.^123^ Absorbance spectroscopy of the enzyme in the presence of either compound confirmed that light was required for inactivation. Spectral features near 379 and 450 nm continuously decreased with increasing light exposure, while a new feature appeared near 320 nm. The decreases at 379 and 450 nm are consistent with reduction of the FAD, while the increase near 320 nm is consistent with covalent modification of the FAD N5. Additional assays showed that the rate of inactivation was much faster with blue light than with either red or green light. The requirement of blue light implicates an excited electronic state of the FAD (FAD*) in the inactivation mechanism.

The proposed mechanism of inactivation (Figure 13B) was inspired by the literature on the photoinduced covalent modification of free^124^ and enzyme-bound flavins,^125–128^ as well as the catalytic mechanism of fatty acid photodecarboxylase.^129,130^ The mechanism begins with the binding of the inactivator to the proline substrate site of the oxidized enzyme prior to exposure to light. Illumination of the enzyme–inhibitor complex with blue light generates an excited flavin state, FAD*. Single-electron transfer from the inhibitor to FAD* generates a FAD semiquinone and either a carboxyl- or sulfur-centered radical. This step reflects the fact that excited flavins are powerful oxidants.^124^ Decarboxylation generates a carbon-centered radical that combines with the FAD semiquinone to form the covalently inactivated enzyme observed in the crystal structures.

## Spermine/Polyamine Oxidases

Spermine oxidase (SMOX) and polyamine oxidase (PAOX) belong to the MAO structural family of flavoprotein amine oxidases and have the same fold as MAO. They catalyze the oxidation of polyamines, such as spermine and spermidine, with PAOXs preferring the *N*-acetylated forms (Figure 14A). The overall reactions generate a shorter polyamine, an aldehyde, and H_2_O_2_. SMOX and PAOX are potential therapeutic targets because their stress-induced activity converts essential polyamines into reactive oxygen species and toxic aldehydes that drive inflammation, DNA damage, and early carcinogenesis.^131–133^ Inhibiting these enzymes potentially offers a strategy to prevent disease progression by suppressing pathogenic oxidative stress without broadly disrupting normal polyamine-dependent cell growth. For example, *N*,*N*′-bis-[buta-2,3-dienyl]-1,4-diaminobutane (MDL72527) is a nonselective covalent inactivator of SMOX and PAOX. The use of MDL72527 in animal models of both colon and gastric cancers led to significant decreases in ROS production, DNA damage, and tumor incidence.^132^

**Figure 14:**
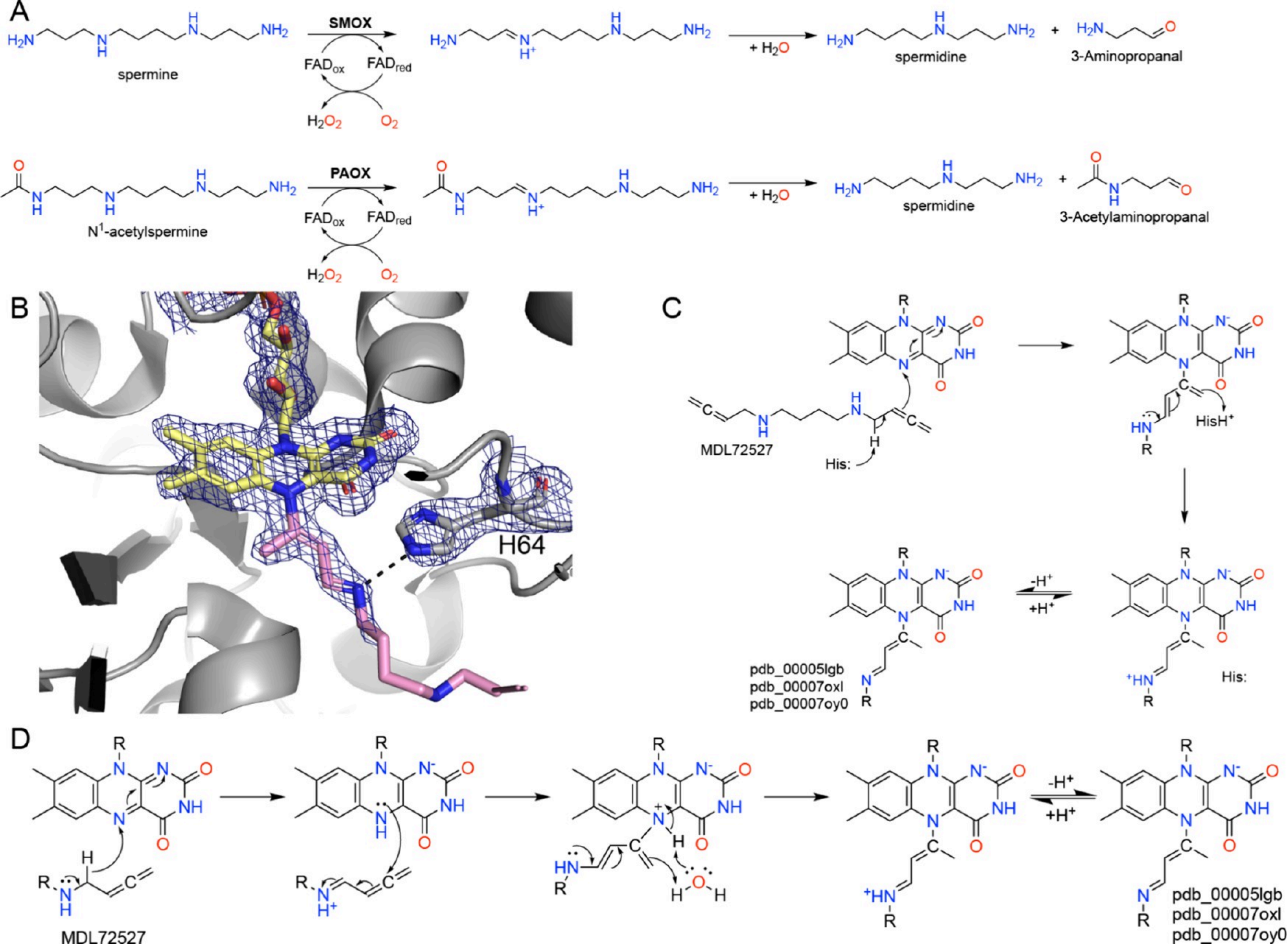
Inactivation of SMOX/PAOX by MDL72527. (A) Reactions catalyzed by SMOX and PAOX. (B) Electron density for the modified FAD in PAOX inactivated by MDL72527 (pdb_00005lgb). The mesh represents a 2*F*_0_-*F*_c_ map (1σ). (C) Proposed mechanism of inactivation based on Wu et al.^134^ (D) Alternative mechanism involving hydride transfer.

The mechanism of action of MDL72527 has been studied. Wu et al. monitored the reaction of murine PAOX with MDL72527 using absorbance spectroscopy.^134^ Inactivation resulted in the appearance of spectral features consistent with the formation of a flavocyanine adduct.^134^ The structures of the covalent adducts of murine PAOX and human SMOX from MDL72527 have been determined (pdb_00005lgb, pdb_00007oxl, pdb_00007oy0).^135,136^ In these structures, the bond to the N5 and the first 6–7 atoms of the adduct have strong electron density, and the rest of the adduct beyond the imine nitrogen presumably is conformationally disordered (Figure 14B).

The proposed inactivation mechanism begins with the nucleophilic attack of the MDL72527 allene on the N5 of the oxidized FAD (Figure 14C).^134^ This step is thought to be facilitated by an active site base that promotes the removal of the amino α-proton of MDL72527. Subsequent electron rearrangements within the adduct and proton transfer back from the protonated active site base result in the proposed flavocyanine adduct. Analysis of the structure of MDL72527-inactivated PAOX suggested that His64 (His62 in human SMOX) could be the active site base (Figure 14B).^135^

An alternative mechanism involving hydride transfer, which does not invoke the active site histidine, is proposed in Figure 14D. Inactivation begins with hydride transfer from MDL72527 to the FAD, generating an iminium species and reduced FAD. Nucleophilic attack by the N5 of FADH^–^ on the activated unsaturated intermediate, followed by water-assisted proton transfer from the N5 to the alkene, generates the conjugated adduct observed crystallographically.

## Cytokinin Oxidase/Dehydrogenase

Cytokinins are phytohormones that consist of adenine or adenosine carrying an *N* ^6^-isoprenoid or aromatic side chain. Cytokinins have wide-ranging effects on plant growth, development, and physiology, and the enhancement of cytokinin levels results in favorable phenotypes, including increased tolerance to drought stress and higher crop yields.^137,138^ One approach to increasing cytokinin levels in plants is to inhibit cytokinin oxidase/dehydrogenase (CKX), the flavoenzyme that catalyzes the degradation of cytokinins to adenine/adenosine and the corresponding side chain aldehyde (Figure 15A).^138^

**Figure 15:**
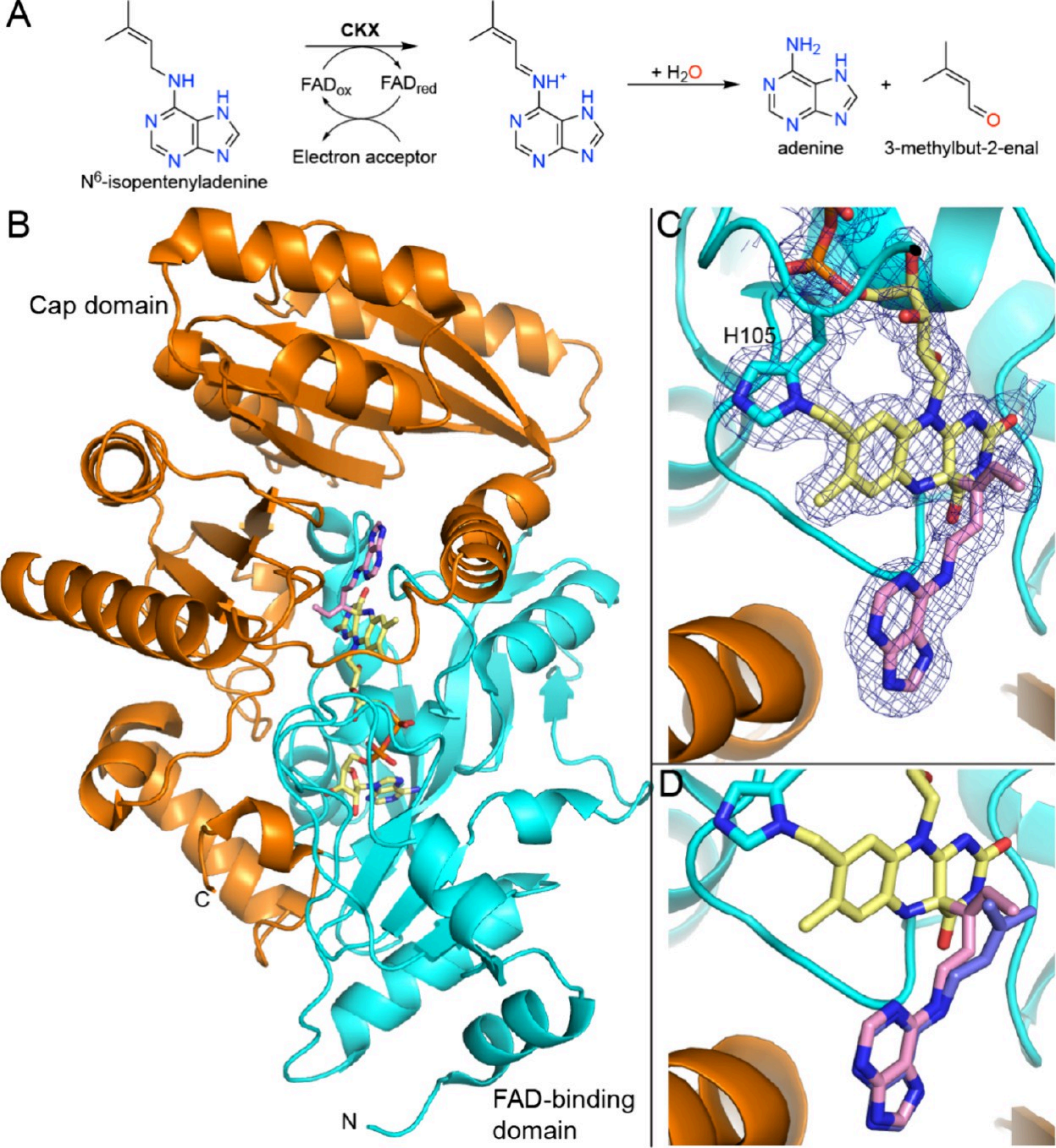
Reaction and structure of CKX. (A) Reaction catalyzed by CKX with *N* ^6^-isopentenyladenine as the substrate. (B) Fold of CKX (pdb_00003bw7). The protein chain is colored according to domains. (C) Structure of CKX inactivated by HA-1 (pdb_00003bw7). The mesh represents a 2*F*_0_-*F*_c_ map (1σ). (D) Comparison of the covalent adduct from HA-1 (pink, pdb_00003bw7) with the imine product of *N* ^6^-isopentenyladenine (blue, pdb_00001w1q).

CKX belongs to the vanillyl-alcohol oxidase flavoprotein family, and its fold comprises an *N*-terminal FAD-binding domain and a C-terminal cap domain (Figure 15B).^139^ The structure of CKX was first determined using the enzyme from *Zea mays*.^140^ Structures of enzyme complexes with biochemically relevant ligands include the imine oxidation product of N^6^-isopentenyladenine (pdb_00001w1q).

The mechanism-based covalent inactivation of CKX by allenic substrate analogs has been explored. Suttle and Mornet demonstrated the promise of *N* ^6^-substituted aminopurine derivatives containing either allenic or acetylenic side chains.^141^ Two allenic compounds, known as HA-1 and HA-8, were found to be time-dependent, irreversible inhibitors, consistent with mechanism-based covalent inactivation. A few years later, Kopečný and co-workers determined the structures of maize CKX inactivated by HA-1 (pdb_00003bw7) and HA-8 (pdb_00003c0p).^142^ The structures revealed the covalent modification of the C4a atom (Figure 15C). The conformation of the adduct is very similar to that of the noncovalently bound imine of *N* ^6^-isopentenyladenine, emphasizing the mimicry of the inactivator to the substrate (Figure 15D). The proposed mechanism of inactivation^142^ begins with oxidation of HA-1/8, producing the reduced FAD and an allenic iminium ion (Scheme 10). Attack of the C4a atom of reduced the FAD on the central carbon atom of the allene group installs the covalent FAD modification.

**Scheme 10:**
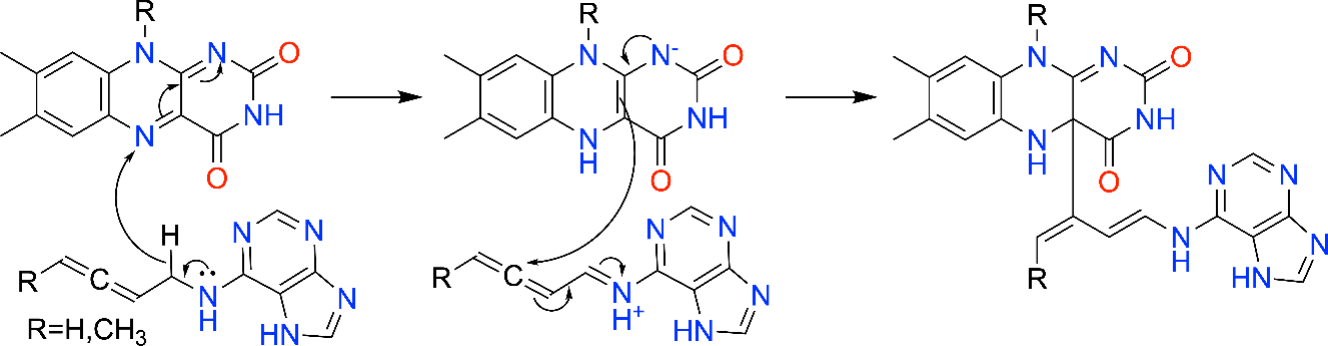
Proposed Mechanism of Inactivation of CKX by allenic Substrate Analogs

## Perspectives

Research on the covalent inactivation of flavin-dependent oxidoreductases spans nearly six decades, evolving from early mechanistic probes, such as sulfite, to contemporary structure-guided drug discovery. Throughout this period, X-ray crystallography has played a decisive role in defining the chemistry of flavin modification. The structural record reveals a limited repertoire of outcomes: N5 alkylation, C4a alkylation, cyclic N5–C4a adducts, and, in select cases, cross-links to protein side chains. In most examples, the modified isoalloxazine adopts a reduced, butterfly-bent conformation, reinforcing the intimate coupling between the redox state and covalent reactivity. These structural snapshots have transformed mechanistic proposals into chemically defined models and have enabled the rational optimization of covalent inhibitors in systems such as MAO and LSD1.

One conclusion from surveying the PDB is the predominance of amine oxidases and dehydrogenases among the structurally characterized cases of covalent flavin inactivation. This distribution is unlikely to be coincidental. Although the central role of amine-oxidizing enzymes in drug discovery has certainly encouraged their structural characterization, flavin-dependent amine oxidases and dehydrogenases may also be particularly prone to irreversible modification because their catalytic cycles intrinsically generate an electrophilic iminium-type intermediate in the proximity of the reduced flavin N5. Covalent inhibition in these systems may be understood as the kinetic diversion of a native catalytic intermediate into a stabilized flavin adduct. Active-site features common to amine oxidases, including hydrophobic cavities, aromatic cages, and restricted solvent access, may further favor productive alignment of the electrophile and the flavin nucleophile. Covalent trapping thus requires no fundamentally new chemistry; it hijacks the reactivity already built into the catalytic mechanism.

These mechanistic features also have implications for drug discovery. Clinical translation has thus far been concentrated in the amine oxidase field, where irreversible MAO inhibitors have achieved therapeutic use, whereas most other structurally characterized covalent flavin inactivators remain preclinical or exploratory.^143,144^ At the same time, covalent flavin trapping is not the only viable strategy for these targets: reversible inhibition is well established in the MAO field, and both covalent and noncovalent LSD1 inhibitors have entered clinical development.^143–146^ Reversible LSD1 inhibition has also shown clinical activity, as illustrated by CC-90011.^147^ The medicinal-chemistry question, therefore, is not whether a flavoenzyme can be inhibited but when covalent diversion of the catalytic mechanism provides sufficient advantage in potency, duration, selectivity, or pharmacology to justify incorporation of a reactive motif. Systematic comparisons with closely matched noncovalent analogues lacking that motif, however, remain relatively rare.

This uneven translational landscape is consistent with a broader mechanistic distinction: most other flavoenzyme classes do not generate electrophiles positioned for attack by N5 or C4a. Dehydrogenases that oxidize C–C or C–O bonds typically produce alkenes or carbonyls that dissociate rather than react further with the cofactor. Monooxygenases channel reactivity through C4a-(hydro)­peroxy or N5-oxide intermediates that are oriented outward toward the substrate rather than inward toward the flavin nucleus. Even noncanonical flavoenzymes that transiently form covalent N5 intermediates generally embed these species within tightly choreographed catalytic cycles that disfavor dead-end adduct formation. Thus, the rarity of non-amine oxidase examples likely reflects intrinsic electronic and geometric constraints governing flavin reactivity rather than a lack of inhibitor exploration.

Looking forward, two complementary opportunities emerge. First, continued structural characterization of covalent flavin adducts, particularly through time-resolved crystallography and cryogenic trapping, will refine our understanding of the intermediate states that border catalysis and inactivation. Second, expanding covalent inhibitor design beyond amine oxidases may require strategies that either introduce engineered electrophiles capable of engaging reduced flavin or exploit cryptic nucleophiles revealed during catalytic cycling. The structural framework assembled here provides a foundation for such efforts and underscores a central lesson: covalent flavin modification is most successful when it coopts, rather than opposes, the intrinsic chemical logic of the enzyme.

## Supplementary Material



## Footnotes

1 Although two structures forcompound S2157 were deposited in the PDB (pdb_00006 kgo, pdb_00006kgp), they derived from a single X-ray diffraction data set and couldhave been modeled in a single structure with occupancy refinement.

## Notes

Spreadsheet listing the PDB entries referred to in this article (XLSX)

## ABBREVIATIONS

2-PCPA: *trans*-2-phenylcyclopropylamine
B32G: but-3-yn-2-ylglycine
C2C: tetrahydrothiophene-2-carboxylate
CPG: *N*-(cyclopropyl)­glycine
CKX: cytokinin oxidase/dehydrogenase
D2C: 1,3-dithiolane-2-carboxylate
GSALDH: l-glutamate-γ-semialdehyde dehydrogenase
HYPDH: hydroxyproline dehydrogenase
KDM1A/LSD1: lysine-specific histone demethylase 1
L-T2C: l-thiazolidine-2-carboxylate
MAO: monoamine oxidase
PRODH: proline dehydrogenase
PutA: proline utilization A
THFA: S-(−)-tetrahydro-2-furoic acid
